# Identification and validation of an 11-kinase signature that predicts chemo- and radiosensitivity in gastric cancer

**DOI:** 10.1016/j.ebiom.2026.106154

**Published:** 2026-02-11

**Authors:** Changyuan Hu, Sung-Young Shin, Yanan Wang, Chenbin Chen, Pei Liu, Rita A. Busuttil, Yunjian Wu, Catriona A. McLean, Hongying Shi, Terry Kwok, Lan K. Nguyen, Jiangning Song, Alex Boussioutas, Xian Shen, Roger J. Daly

**Affiliations:** a Wenzhou Medical University-Monash BDI Alliance in Clinical and Experimental Biomedicine, The First Affiliated Hospital of Wenzhou Medical University, Wenzhou, China; b Cancer Program, Biomedicine Discovery Institute, Monash University, Clayton, Australia; c Department of Biochemistry and Molecular Biology, Monash University, Clayton, Australia; d Computational Systems Oncology Program, South Australian ImmunoGENomics Cancer Institute (SAiGENCI), The University of Adelaide, Adelaide, SA, 5005, Australia; e Burning Rock Biotech, Guangzhou, Guangdong, China; f Department of General Surgery, The First Affiliated Hospital of Wenzhou Medical University, Wenzhou, Zhejiang, China; g Zhejiang Province Key Laboratory for Intelligent+ Cancer Biomarker Research and Translation, The First Affiliated Hospital of Wenzhou Medical University, Wenzhou, Zhejiang, China; h School of Big Data & Software Engineering, Chongqing University, Chongqing, China; i School of Translational Medicine, Monash University, 99 Commercial Rd, Melbourne, VIC, 3004, Australia; j Department of Gastroenterology, The Alfred Hospital, 55 Commercial Rd, Melbourne, VIC, 3004, Australia; k Olivia Newton-John Cancer Research Institute, Heidelberg, VIC, Australia; l School of Cancer Medicine, La Trobe University, Bundoora, VIC, Australia; m Anatomical Pathology, Alfred Health and School of Translational Medicine, Monash University, Australia; n Department of Epidemiology and Health Statistics, School of Public Health, Wenzhou Medical University, Wenzhou, Zhejiang, China; o Infection and Immunity Program, Monash Biomedicine Discovery Institute, Monash University, Clayton, Australia; p Department of Microbiology, Monash University, Clayton, Australia; q Australian Research Council Centre of Excellence for the Mathematical Analysis of Cellular Systems (MACSYS), Australia

**Keywords:** Gastric cancer, Chemoresponse, Radioresponse, Biomarker, Machine learning

## Abstract

**Background:**

Most gastric cancer (GC) patients receive uniform treatment due to the lack of predictive biomarkers for chemotherapy or radiotherapy. We previously identified epithelial-mesenchymal transition (EMT) and metabolism subgroups in GC cell lines based on kinomic profiles, but their clinical relevance was unknown.

**Methods:**

We developed an ensemble model using 37 kinases that differed between cell line subgroups to classify stage II-IV GC into EMT and metabolism subgroups. Survival differences between those who received chemotherapy or radiotherapy and those who did not were compared within each subgroup to validate the model effectiveness in predicting therapeutic response. An iteration approach was further applied to optimise and validate the feature set via multiple publicly-available datasets.

**Findings:**

An 11-kinase signature stratified 893 patients into two subgroups. The metabolism subgroup showed significantly better survival with chemotherapy (HR_multivariable_ = 0.56) and radiotherapy (HR_multivariable_ = 0.55), whereas no such improvement was observed in the EMT subgroup. Significant interaction between kinomic subgroups and treatments were noted. The chemotherapy benefits between subgroups were greater with 5-fluorouracil-based regimens than cisplatin-based ones. This kinomic taxonomy was distinct from Lauren classification and previous transcriptomic subtypes and also suggested differential therapeutic vulnerabilities between subgroups.

**Interpretation:**

This model holds promise for optimising chemotherapy and radiotherapy decisions for GC.

**Funding:**

Biomedicine Discovery Scholarship and Graduate Research Completion Award, Monash University; 10.13039/501100001809National Natural Science Foundation of China (81602165) (C.H.). Australian Research Council Centre of Excellence for the Mathematical Analysis of Cellular Systems (CE230100001) (L.K.N.).

**Research in context**

**Evidence before this study**

Chemotherapy and radiotherapy are the cornerstone treatments for gastric cancer (GC). However, due to the inherent heterogeneity of GC, treatment outcomes vary widely. While the CLASSIC trial demonstrated that adjuvant chemotherapy improves prognosis, nevertheless, nearly half of the patients who did not receive chemotherapy survived for more than five years. Meanwhile, the ARTIST trial and the Intergroup-0116 study showed that while radiotherapy did not benefit all individuals with GC, it was effective for those with lymph node metastasis or who had undergone D1 gastrectomy. These findings underscore the need for predictive biomarkers that can accurately stratify patients, guide treatment decisions, and minimise unnecessary interventions. We searched PubMed (https://pubmed.ncbi.nlm.nih.gov/) for studies published between 1 January 2010 and 31 December 2023 using the terms: “gastric cancer” AND “chemotherapy response OR radiotherapy response” AND “biomarker OR signature”. Currently, no biomarker studies exist that can simultaneously stratify patients based on their responses to both chemotherapy and radiotherapy. While some preclinical studies on predictive biomarkers can stratify patients based on either chemosensitivity or radiosensitivity individually, the vast majority lack a multi-cohort validated system to effectively identify treatment beneficiaries (PMID: 29725014, 34376661, 28902346).

**Added value of this study**

In this retrospective multicohort study, we developed an 11-kinase signature that stratified patients into two subgroups: the metabolic subgroup showed significantly better survival after chemotherapy (HR 0.56) or radiotherapy (HR 0.55), effects not observed in the EMT subgroup. The benefits of chemotherapy between subgroups were more pronounced with 5-fluorouracil-based regimens than cisplatin-based ones. Our taxonomy, which differs from the Lauren subclassification and the previous definition of metabolic and mesenchymal subtypes, also suggested alternative treatment approaches: immunotherapy for the EMT subgroup and EGFR or ERK inhibitors for the metabolic subgroup.

**Implications of all the available evidence**

Clinical development of this signature may effectively stratify treatment response to radiotherapy and chemotherapy, which would promote personalised medicine for GC.

## Introduction

Gastric cancer (GC) ranks fifth globally in both incidence and mortality across all cancers, with over half of new cases diagnosed in the Asia Pacific region.^1^ In the United States alone, there were an estimated 26,380 new cases of GC in 2022, with about 11,090 cancer-related deaths expected,^2^ presenting a grim reality with a 35.7% 5-year overall survival (OS) rate.^3^ The standard care for locally advanced-stage GC includes curative-intent surgery with D2 (extended) lymph node dissection and adjuvant chemotherapy.^4^ Additionally, radiotherapy, immunotherapy, and targeted therapy have all shown effectiveness in treating GC, but are limited by their efficacy, objective response rate and targetable populations.^5^, ^6^, ^7^

Currently, the management of GC through surgery, chemotherapy (including 5-fluorouracil (5-FU), paclitaxel and platinum-based regimens), and radiotherapy is predominantly guided by clinical assessments of surgical resectability, TNM staging and patient factors that may limit treatment options.^8^ However, due to the heterogeneous nature of this disease, this approach often leads to varied treatment outcomes. For instance, while the CLASSIC trial demonstrated an improved prognosis with adjuvant chemotherapy following D2 radical gastrectomy compared to surgery alone, approximately half of the patients who abstained from chemotherapy were still alive after five years, highlighting the need for improved patient stratification that avoids unnecessary treatment.^4^ The efficacy of adjuvant radiotherapy in GC remains unclear. The ARTIST trial found that adjuvant chemoradiotherapy (CRT) was beneficial for patients with lymph node metastasis,^9^ whereas a per-protocol analysis of the CRITICS trial revealed significantly worse OS outcomes with adjuvant CRT compared to chemotherapy alone.^10^ Furthermore, the ARTIST-II trial indicated no survival difference between adjuvant CRT and chemotherapy in cases where a platinum-based combination regimen was administered.^11^ Given the majority of GC treatment guidelines continue to adopt a “one-size-fits-all” approach for chemotherapy and/or radiotherapy,^8^^,^^12^ there is an urgent need for methods capable of stratifying the heterogeneous population of patients with GC into homogeneous subtypes, that more predictably respond to specific therapies thereby avoiding unnecessary toxicities and futile therapy.

To date, GC classifications based on histomorphology features are extensively utilised in clinical settings. One such subgrouping is the Lauren classification, where patients with diffuse type GC typically exhibit poorer prognosis and less favourable treatment responses.^13^ Unfortunately, these histopathological features are insufficient for guiding extensive clinical decision-making, as they frequently lack precision and require large cohorts to demonstrate clinical significance.^13^ With the advent of high-throughput technologies, GC molecular subgroupings have gained prominence over the last decade. The Cancer Genome Atlas (TCGA) project, for instance, characterised GC into four clusters based on genomic data: Epstein–Barr virus (EBV)-positive, microsatellite instability (MSI), genomically stable (GS) and chromosomal instability (CIN).^14^ A proteomic clustering system identified three subgroups of GC, each linked to specific biological processes: cell cycle, immune response and EMT.^15^ Very recently, we proposed a kinomic subclassification on GC cell lines with distinct molecular features and drug sensitivity between epithelial-mesenchymal transition (EMT) and metabolism subgroups.^16^ Despite these sophisticated and comprehensive studies providing an extensive understanding of GC molecular subtypes, their direct impact on improving prognosis and guiding clinical practice remains limited.

Encouragingly, biomarkers developed by statistical model or machine learning (ML) have excellent utility across a wide range of cancers when combined with multi-omics data.^17^ Based on gene expression data, an 18-probe signature (ColoPrint) developed by the Cox proportional hazards model was superior to the American Society of Clinical Oncology staging system in assessing cancer recurrence risk of stage II and III colorectal cancer.^18^ An FDA-approved molecular signature, termed the Prosigna™ Breast Cancer Prognostic Gene Signature Assay (PAM50 test), predicts response to anthracycline chemotherapy in node-positive breast cancer.^19^ Similarly, numerous studies have explored biomarkers for treatment response in GC.^20^^,^^21^ Among these, a classifier composed of four genes effectively stratifies patients with resectable, stage II–III GC for both prognosis and response to adjuvant chemotherapy.^22^ However, while HER2 and MSI status can guide HER2-targeted treatment and immunotherapy in GC, respectively,^8^ to our knowledge there are limited guidelines recommending use of specific biomarkers to stratify patients for particular chemotherapeutic regimens. Furthermore, amongst preclinical studies on predictive biomarkers for GC radiotherapy, a multi-cohort validated system for effectively selecting radiotherapy beneficiaries is also lacking.

In this study, we built upon our previous (phospho)proteome-based definition of EMT and metabolism GC subtypes^16^ and initially developed a predictive model using 37 kinase proteins to distinguish the two subtypes, characterised by differential chemosensitivity, in patient cohorts. Subsequent optimisation of the model's features and validation across multiple cohorts led to the creation of an 11-kinase signature. This work developed a kinase signature capable of predicting both chemotherapy and radiotherapy responses in GC, designed and reported according to the TRIPOD guidelines.^23^ Additionally, our investigation addresses differential molecular characteristics and responsiveness to immunotherapy and specific targeted treatments across the two kinomic subgroups.

## Methods

### Cell line proteomic datasets

The internal training and validation datasets, derived from our in-house proteomics data on GC cells, are detailed in our previous work,^16^ including sample preparation, and data processing. Details on cell line identity, authentication and culture conditions are also provided. A combined kinome dataset was retrieved from the KinMap, Uniprot, ENZYME, PhosphoSitePlus, and WikiKinome databases. Of note, although our previous study identified 40 differentially expressed kinases between two subgroups of GC cell lines, three of these kinases were not identified across other external validation datasets. Therefore, in this study, we utilised the remaining 37 kinases to construct the dataset, which is represented by a 42 × 37 matrix, encompassing 14 distinct GC cell lines, each with three biological replicates. The 42 cellular samples were divided into internal training and validation cohorts.

### Patient validation cohorts

Participants across all external validation cohorts were further screened based on the following criteria: 1. Patients with a TNM stage of I or with an unspecified stage were excluded; 2. Those annotated as having undergone adjuvant, neoadjuvant, or palliative chemotherapy and/or radiotherapy were incorporated into the corresponding clinical effectiveness analysis. However, cohorts where treatment labels were uniform across all their patients, such as cohorts where everyone underwent chemotherapy or no-radiotherapy, were not included in the corresponding analysis. This exclusion is due to the inability to compare survival benefits in cohorts that lack either treatment-positive or negative groups; 3. For some clinical cohorts with detailed chemotherapy regimen information, participants were categorised based on their treatment plans into groups of chemotherapy (−), cisplatin (+) (potentially including 5-FU), and 5-FU (+)/cisplatin (−) (explicitly excluding cisplatin), to analyse the benefits of different chemotherapy regimens.

The external validation cohorts are detailed in Table S1.

PUCH cohort: The Peking University Cancer Hospital (PUCH) cohort is a proteomics dataset from 84 patients with diffuse GC, who received gastrectomy from November 2012 to July 2015 and ended follow-up from January 2013 to January 2017.^15^ We extracted the matrix containing the abundance of the 37 kinase proteins from the original proteome data provided by the authors for further analysis.

Fudan cohort: The Fudan cohort is a proteomics dataset derived from formalin-fixed, paraffin-embedded tumour tissues of 206 patients with advanced GC prior to receiving chemotherapy or targeted therapy.^24^ The initial diagnosis date ranged from January 2002 to March 2020. Tumour response to neo-adjuvant drug treatment was recorded as a complete response (CR), partial response (PR), stable disease (SD), or progressive disease (PD). We extracted the matrix containing the abundance of the 37 kinase proteins from the original proteome data provided by the authors for further analysis.

TCGA cohort: In terms of the TCGA-STAD (The Cancer Genome Atlas Stomach Adenocarcinoma, simplified as TCGA in the following text) cohort,^14^ the mRNA HTSeq Counts files were downloaded from the data portal (https://portal.gdc.cancer.gov/), which comprises 375 GC specimens. The initial diagnosis date ranged from 1996 to 2013, and last follow-up ended between 2011 and 2013. These raw count files were normalised based on transcripts per million and log2 transformed before extracting the RNA expression matrix of 37 kinases for further analysis.

ACRG/Australian/KUGH/KUCM/MDACC/Singapore cohorts: Gene expression profiling data of GC tissues were downloaded from the GEO (http://www.ncbi.nlm.nih.gov/geo/) database to validate the predictive model, including: GSE66229 (Asian Cancer Research Group (ACRG) cohort,^25^ n = 300, operation conducted from December 2004 to May 2008 and last follow-up ended from May 2004 to October 2010); GSE35809 (Australian cohort, n = 68, a subset of the broader GSE51105 series,^26^ operation conducted from October 1995 to August 1999 and last follow-up ended from June 1996 to August 2004); GSE26899 (Kosin University Gospel Hospital (KUGH) cohort,^21^ n = 93; operation conducted from 1999 to 2006); GSE26901 (Kosin University College of Medicine (KUCM) cohort,^21^ n = 109, operation conducted from 1999 to 2006); GSE28541 (University of Texas MD Anderson Cancer Center (MDACC) cohort, n = 40, sample collected from 2002 to 2010); and GSE15460 (Singapore cohort,^27^ n = 201). All gene expression datasets were normalised and log 2 transformed before extracting the 37-kinase matrix for further analysis.

A total of 893 patients from seven independent cohorts (all of the above cohorts with the exception of the Fudan cohort and Singapore cohort) were utilised for validation. Of these, 586 patients were male and 307 were female (median age, 63 years; range, 24–90 years); sex data were obtained from the source datasets, although the method of ascertainment was not reported. A total of 695 cases had TNM staging II-III and 198 were stage IV. Regarding treatment, 853 subjects from six cohorts were included for chemoresponse analysis, with 463 subjects receiving chemotherapy, and 644 subjects from four cohorts were screened for radioresponse analysis, with 172 subjects having radiotherapy. No HER2 expression status or anti-HER2 treatment were recorded in these cohorts. The median OS was 47.70 months, and clinical characteristics of the patients and distribution of missing data is shown in Figure S1. The Singapore cohort was utilised for subgroup comparisons. The Fudan cohort was utilised for evaluating ERBB2 expression and anti-ERBB2 treatment response across kinomic subgroups.

The genomic alterations data of the TCGA cohort were obtained from cBioPortal.^28^ Drug responses (the area under the fitted dose response curve, AUC) of subset-specific GC cell lines were downloaded from the genomics of drug sensitivity in cancer (GDSC) database.^29^ The subgrouping of GC cell lines in the GDSC dataset has been explored in our previous work.^16^

### Model training and scoring feature importance

All cohorts analysed using kinase matrix were firstly normalised by z-score before model training and validation. The cellular proteomics data with EMT or metabolism labels^16^ were randomly split into a training cohort and an internal validation cohort at an 8:2 ratio,^30^ and then trained using the random forest (RF) algorithm (Python 3.0 ‘*scikit-learn*’ package, number of estimator = 100). For the accuracy of the internal validation set, we used the following commonly used metrics and measures, including Accuracy, F1 score and receiver-operating characteristic curve with the corresponding area under the ROC curve (AUC). These performance metrics are defined by the following equations:

Accuracy=(TP+TN)/(TP+TN+FP+FN)

F1score=2TP/(2TP+FP+FN)

Here, TP is True Positive, TN is True Negative, FP is False Positive, and FN is False Negative.

The RF model was then applied to classify GC patients from clinical cohorts into either the EMT subgroup or metabolism subgroup using the 37-kinase profiling data. To achieve robust prediction, we trained the 500 RF models by randomly sampling training data, these models made predictions on a given dataset, and the final prediction is determined based on a majority vote (>60%). To calculate the feature importance, we averaged the importance scores of 500 RF models (based on the built-in function of RF) and sorted the features by the score.

### Sample size and missing data

We used publicly available genomic and proteomic cohorts to analyse signatures. With that in mind, we estimated the required sample size assuming a two-sided α of 0.05, 80% power, an anticipated hazard ratio (HR) of 0.60 for treatment response, a 5-year OS of 30%, and an allocation of 40% to the chemotherapy arm. The unadjusted sample size (N_0_) was first computed using the standard Cox-PH two-group formula.^31^, ^32^, ^33^, ^34^, ^35^, ^36^, ^37^ We then inflated this by dividing by (1 – attrition) to allow for 30% attrition,^38^^,^^39^ yielding a final required sample size of 256 patients per subgroup. This number increases to 384 when focusing on the radioresponse with about 20% of the total cases receiving radiotherapy.^33^^,^^40^

In this study, the predictor datasets used for ML model development had no missing values in the training and most validation cohorts, except for the PUCH and Fudan cohorts. As proteomic datasets, these two cohorts naturally exhibited more missing values than transcriptomic data, with a median missing rate of 4.76% per predictor (range: 0%–74.81%) and 99.31% samples having at least one missing value. We used the imputed data provided by the original study, which applied minimum-value imputation.^15^^,^^24^ For the clinical variables used in regression models, the missing data rates were 2.25% for TNM staging, 5.06% for Lauren classification, and 5.44% for tumour location, with 9.00% of participants missing at least one predictor. The high missing rates for Lauren classification and tumour location were primarily driven by the MDACC cohort, which did not report these two variables. Because this mechanism clearly violates the Missing-At-Random assumption, we employed a complete-case analysis approach for all regression models, deliberately avoiding any imputation.

### Outcomes and risk groups

The outcome of the predictive model is the categorisation of patients with GC into two subgroups. After extensive studies based on our own study^16^ and other transcriptome studies,^27^^,^^41^ we hypothesised that metabolism and EMT tumours would be more and less responsive, respectively, to conventional therapeutic strategies of GC. In applying a ML approach, since patients cannot be formally labelled EMT and metabolism, we adopted this differential responsiveness as a validation metric reflected by HR and statistical significance (P value) between treated and surgery-only patients within each subgroup. For instance, if the model properly stratified the cohorts, chemotherapy or radiotherapy would be a protective factor (low-risk group) in the metabolism subgroup, but not in the EMT one. The outcome of the survival analysis was OS, measured for each participant from the date of surgery (origin/start time) until the date of death from any cause (end time), or the last follow-up, whichever occurred first. Participants who were alive at the last follow-up were censored at that time point. HR estimated from the Cox model were interpreted as an incremental decrease in the death in the treated group relative to non-treated group. This method evaluates the model performance by determining whether there is a clinically meaningful stratification at the patient level. Specifically, after subtyping by the cell classifier, the sensitive group is expected to show a statistical improvement in prognosis following the appropriate treatment, while the resistant group should not exhibit significant survival benefits regardless of treatment.

### Predictor simplification and model validation

The desirable model (37-kinase signature) with its feature importance was selected for further optimisation based on two criteria: a) the HR for the EMT subgroup was greater than that for the metabolism subgroup; b) P values were less than 0.05 in the metabolism subgroup and greater than 0.05 in the EMT subgroup after survival analysis. To refine the kinase signature, we devised and implemented the following procedures: Step 1) Evaluate the importance score of individual 37 features based on desirable RF model; Step 2) Start adding the feature having the higher importance score in the training data; Step 3) Train the model using GC cell line proteomics data; Step 4) Stratify the patient cohorts into the EMT and metabolism subgroups based on the trained model; Step 5) Conduct an univariable analysis against chemoresponse; Step 6) Check the chemosensitivity of two patient subgroups, with the underlying assumption: a) The EMT group is expected to have a higher HR than the metabolism; b) the metabolism subgroup should have significant chemo-sensitivity (P < 0.05), while the EMT subgroup shows no significant difference (P > 0.05). We repeated Step 2–6 of this procedure against all 37 features. Ultimately, the model with the fewest number of kinases that still produced a P value greater than 0.05 in the EMT subgroup and less than 0.05 in the metabolism subgroup was chosen for external validation.

### PD-L1 evaluation

Immunohistochemistry (IHC) was performed on tissue microarrays comprising 80 GC samples corresponding to those in the GSE51105 dataset^26^ to assess PD-L1 expression status, as previously described.^42^ Briefly, IHC staining was performed using the anti-PD-L1 antibody clone E1L3 N (Cell Signaling Technology, Danvers, USA, RRID: AB_2687655) at 1:400 dilution on the Leica Bond Max staining platform (Leica Biotechnologies, Wetzlar, Germany). The antibody was validated using Dako-supplied, pre-validated cell line controls, followed by in-house confirmation with tonsil tissue and tumour samples of known PD-L1 status. The protocol undergoes biannual assessment through external quality assurance programs. Detection was undertaken using EnVision FLEX, High pH system on the Dako Omnis platform (Agilent Technologies, Santa Clara, USA). The PD-L1 was calibrated to match the staining patterns of the Dako PD-L1 IHC 22C3 pharmDx assay (Agilent Technologies) and validated by external quality assessment (QuIP GmbH, Berlin, Germany). Scoring was performed according to the GC guidelines for the Dako 22C3 pharmDx assay (Agilent Technologies) using the Combined Positive Score (CPS), as described by the manufacturer. A CPS score of >1 was considered as expressing PD-L1.

### Ethics

Written informed consent was obtained and ethical approval was acquired from the Institutional Review Boards of the individual hospitals that participated in the study (Human Research Ethics Committee, Peter MacCallum Cancer Centre, Melbourne, Australia, HREC ref 2005.075 and 12/25).

### Statistics

The median survival time and its 95% CI were estimated based on the Kaplan–Meier survival curve. Log-rank and Cox proportional hazards models were used for survival analyses of OS by R package ‘*survival*’. Age was treated as continuous variable, while sex, TNM staging, Lauren classification, tumour location, chemotherapy and radiotherapy were treated as factors.

The key assumptions of the Cox proportional hazards model, including the proportional hazards (PH) assumption and the linearity of continuous predictors, were formally evaluated. The PH assumption for all covariates was assessed by examining the scaled Schoenfeld residuals. Linearity for the continuous predictor, age, was assessed by comparing the fit of the standard linear model to a more flexible model using a 3-knot restricted cubic spline, with the comparison evaluated via a likelihood-ratio test. Univariable Cox regression analyses were conducted to evaluate the association between each clinical variable and OS, and to assess additive and multiplicative interactions, evaluating whether treatment effects varied by subgroup. The relative excess risk due to interaction, along with 95% CI and P values, was estimated using the delta method via the R package ‘*interactionR*’. Variables identified as relevant based on both univariable analysis and clinical judgment were retained for multivariable modelling. To evaluate the variations in continuous variables across two GC subgroups, we reported their mean and standard deviation. Variables that satisfied both the normality assumption (Shapiro–Wilk P ≥ 0.05) and equal variances (Levene's P ≥ 0.05) were compared with the independent two-sample Student's t-test. Variables that violated either assumption or displayed notable outliers were analysed with the nonparametric Wilcoxon rank-sum test. Regarding categorical (factor) variables, their frequencies were detailed, and were analysed by first inspecting the expected cell counts in each contingency table. If all expected counts were ≥5, we applied the two-sided Pearson χ^2^ test; if any expected count was <5, we used the two-sided Fisher's exact test. All tests were two-sided, with α = 0.05. Drug response on tissue samples were predicted by R package ‘*oncoPredict*’.^43^ Drug sensitivity data in cell lines were mined from the Cancer Cell Line Encyclopedia and compared between kinomic subgroups using AUC values. Immune infiltration and immunotherapy benefit were performed by R package ‘*GSVA*’ and ‘*ESTIMATE’*, and subclass mapping (SubMap) module from GenePattern, respectively. The SubMap analysis involves multiple pairwise comparisons between kinomic subgroups and immunotherapy responses. To control for the increased risk of Type I errors from these multiple tests, we applied a Bonferroni correction. This was performed by multiplying each raw P value by the total number of comparisons made. An adjusted P value of <0.05 was then considered statistically significant, as is standard for this conservative correction method.

For statistical analyses of profiling data, an FDR-corrected P value (adjusted P) < 0.05 was adopted as statistically significant in the most cases, but some other thresholds were also considered according to the recommendation of the specific high-throughput statistical methods, such as FDR <0.25 was adopted in gene set enrichment analysis (GSEA). For all statistical analyses of clinical data, a P value < 0.05 was considered as statistically significant.

### Role of funders

Funding sources for this study had no role in study design; collection, analysis, and interpretation of data; writing of the report; and decision to submit the paper for publication.

## Results

### Development of a machine learning pipeline for predictive biomarker identification

**Fig. 1:**
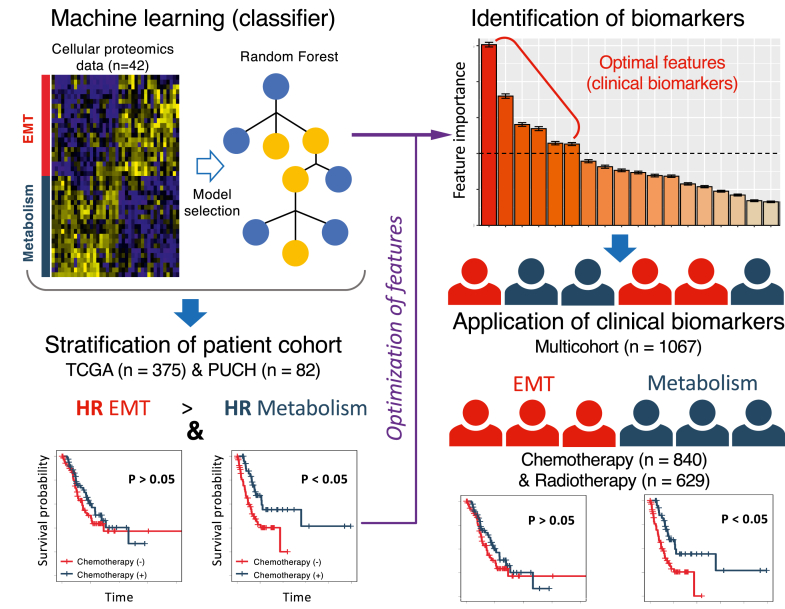
**Workflow of 11-kinase signature prediction and validation**. Schematic workflow of machine learning, feature simplification and validation utilising cellular and clinical datasets of gastric cancer (GC). For the application of clinical biomarkers to chemotherapy we utilised the The Cancer Genome Atlas (TCGA), Asian Cancer Research Group (ACRG), Kosin University Gospel Hospital (KUGH), Kosin University College of Medicine (KUCM), Peking University Cancer Hospital (PUCH), and Australian cohorts. For the application to radiotherapy, we used TCGA, ACRG, University of Texas MD Anderson Cancer Center (MDACC), and Australian cohorts.

There is an urgent clinical need for predictive biomarkers that can stratify patients with GC into optimal treatment regimens. However, the predictive accuracy and utility of biomarkers derived from preclinical models are limited by discrepancies between *in vitro* and clinical datasets, as well as disease heterogeneity.^44^ In this study, we develop an innovative ML-based biomarker identification pipeline to overcome these problems and use it to stratify GC cohorts. The pipeline comprises four primary components: (i) supervised ML, (ii) patient cohort sub-grouping, (iii) feature selection and optimisation, (iv) validation of predictive biomarkers (Fig. 1). Specifically, the study builds upon our previous identification of 40 kinases that are differentially-expressed between two subgroups of GC cell lines, designated EMT and metabolism.^16^ First, we train a RF classifier based on the expression profile of these kinases to predict EMT and metabolism cell lines and the same subgroups of GC tumours. Then, we further refine the model according to the contribution of each feature (kinase). Finally, we explore the treatment benefits and molecular characteristics of the two GC subgroups classified by a simplified 11-kinase signature.

### Identification of a 37-kinase signature that stratifies chemoresponse

**Fig. 2:**
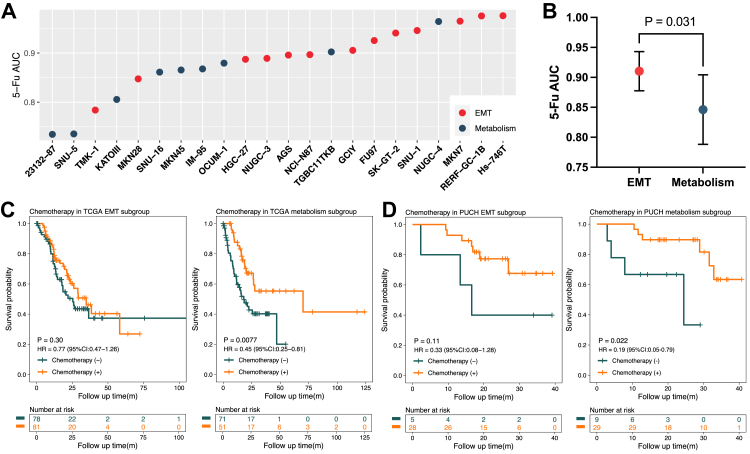
**A kinase signature stratifies gastric cancer patients into subgroups with contrasting chemosensitivity**. **A** Responses of GC cell line subgroups to 5-Fluorouracil (5-FU) in the Cancer Cell Line Encyclopedia. Epithelial-mesenchymal transition (EMT) and metabolism GC cell lines are indicated and exhibited a significant difference in drug sensitivity (indicated by area under curve, AUC). **B** A statistical analysis of 5-FU sensitivity between EMT and metabolism GC cell lines (student t-test). Data are presented as mean ± 95% confidence intervals (CI). **C** Chemosensitivity of the 2 subgroups in the The Cancer Genome Atlas (TCGA) cohort. The cohort was split into epithelial-mesenchymal transition (EMT, left) and metabolism (right) subgroups using the 37-kinase signature. The survival difference, hazard ratios (HRs), 95% CI between chemotherapy (+) and chemotherapy (−) in each subgroup were evaluated by univariable Cox proportional hazards regression analysis. **D** Chemosensitivity of the 2 subgroups in the Peking University Cancer Hospital (PUCH) cohort. This cohort, consisting exclusively of diffuse GC, was split into EMT (left) and metabolism (right) subgroups using the 37-kinase signature. The survival difference, HRs and 95% CI between chemotherapy (+) and chemotherapy (−) in each subgroup were evaluated by univariable Cox analysis.

Previously, we identified 40 kinases that are differentially-expressed between EMT and metabolism subgroup GC cell lines.^16^ Since 3 of these kinases were not detected in certain validation cohorts, we reduced this signature to 37 kinases. In the earlier study, GC cell lines from the GDSC database were categorised into EMT and metabolism subgroups on the basis of the kinase signature, with the two subgroups exhibiting differential responses to certain small-molecule targeted drugs.^16^ Here, we specifically focus on the differential response to 5-FU, a backbone chemotherapeutic agent for GC.^8^^,^^12^ Notably, the sensitivity to 5-FU (indicated by a lower AUC) was significantly greater in the metabolism cell lines compared to the EMT subgroup (P = 0.031, student t-test, Fig. 2A and B). To develop this signature as a predictive biomarker, the next step was to train a kinase-based classifier for application on clinical GC tissue samples. We employed a RF classifier as our base ML model, utilised the cellular proteome data of the kinases as input data (Table S2),^16^ and categorised the EMT and metabolism subgroups as target data. Our RF model accurately predicted the subclass of the cancer cell lines by utilising 37 features (kinases) with accuracy, F1 score, and AUC all exceeding 99.5% (Figure S2A, see Methods).

To assess the potential clinical relevance of our feature set, we applied it to external clinical validation datasets. Despite the expression data for the 37 kinases across 8 validation cohorts (see Methods) originating from varied sequencing techniques and biological backgrounds, such as RNA sequencing, proteomics, gene chips, cell lines, and tissue samples from diverse ethnic groups, the z-score normalisation process resulted in no apparent outlier cohorts upon principal component analysis, suggesting the applicability of our training and validation data across platforms and biological backgrounds (Figure S2B). We categorised the TCGA cohort into EMT and metabolism subgroups using our trained RF model with 37 features at the transcriptomic level. Since the feature set effectively subdivided the TCGA cohort into two subgroups (Figure S2C), we evaluated the predictive power of the features by analysing response to chemotherapy. As illustrated in Fig. 2C, there was no significant survival difference between chemotherapy and non-chemotherapy treatments in the EMT subgroup of the TCGA cohort (P = 0.30, HR = 0.77), while in the metabolism subgroup, patients who received chemotherapy exhibited a significantly better OS compared to those who did not (P = 0.0077, HR = 0.45).

In light of a recent meta-analysis indicating that chemotherapy is beneficial for the Lauren intestinal GC subtype but not the diffuse one,^13^ we compared subclassification using Lauren subtypes and our prediction model using the TCGA cohort. The resulting groupings were not significantly related (P = 0.18; Chi-square test; Figure S2D; Table S3) indicating that our taxonomy did not simply recapitulate the Lauren classification and represented a novel approach. Furthermore, application of the Lauren classification to the TCGA cohort indicated that chemotherapy provides significant survival benefits for both the diffuse (P = 0.011, HR = 0.4) and intestinal (P = 0.0042, HR = 0.49) subtypes (Figure S2E). To further emphasise the power of our taxonomy, we applied it to the PUCH cohort consisting solely of diffuse GC. Within this cohort, the metabolism subtype still responded well to chemotherapy (P = 0.022, HR = 0.19), but the patients with EMT tumours did not benefit after cytotoxic treatment (P = 0.11, HR = 0.33; Fig. 2D). Taken together, these data suggested that the 37-kinase signature represented a new stratification approach for GC chemotherapy.

### Development of a simplified 11-kinase signature for stratifying chemoresponse

**Fig. 3:**
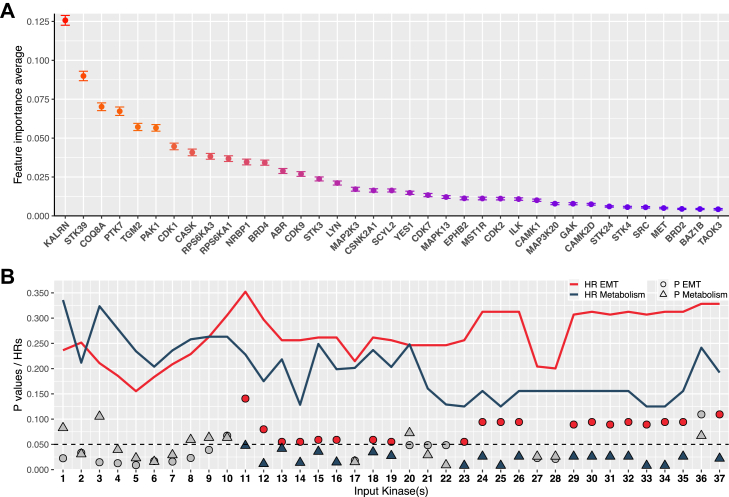
**Simplification of the kinase signature**. **A** Average feature importance scores of the 37 input kinases, with error bars representing 95% confidence intervals of the mean from 500 iterations of model training. **B** Performance of 37 predictive models with different number of input kinases. The model numbers, corresponding to the number of kinases used in model construction, were arranged in ascending order along the x-axis. The y-axis indicates the P values or HRs of each kinomic subgroup from univariable analysis when predicting patient responses to chemotherapy. Red and dark blue shading highlight input kinase with P values greater than 0.05 for the epithelial-mesenchymal transition (EMT) subgroup but less than 0.05 for the metabolism subgroup.

In order to optimise the feature set and enhance practical utility we followed the procedure of predictor simplification (see Methods) against all 37 features and used the PUCH cohort as a patient cohort for stratification. In brief, we optimised the features by retaining the minimum number of features necessary to maximise the HRs difference between the kinomic subgroups, while ensuring that the P value for the metabolism subgroup is less than 0.05 but it exceeds 0.05 for the EMT subgroup. When the features with higher importance score were sequentially added (Fig. 3A), the model performance on the internal validation set increased and then plateaued after including the top five kinases, surpassing 0.995 (Figure S3A). When applied to the PUCH cohort, as the predictive model incorporated an increasing number of features, its proficiency in forecasting chemosensitivity markedly improved. Once the model incorporated ≥10 features, regardless of the total number used in its training, there were predominantly higher HRs and P values when comparing chemoresponse in the EMT subgroups to that in the metabolism subgroups (Fig. 3B). This trend underscored the limited responsiveness of EMT tumours to 5-FU than metabolism tumours. Starting with the model comprising 11 features (total importance score of these features exceeded 60%), the chemoresponse stratification of the model was similar to the final model having 37 features, thus we proposed the top 11 features as a simplified predictive signature (Fig. 3B). However, the definition of a 37-kinase signature provides flexibility if certain kinases are not detected by particular profiling platforms. Application of this simplified signature to stratification of the PUCH cohort is shown in Figure S3B and the expression profile of the 11 kinases in this cohort is compared to the cell line panel in Figure S3C.

Of note, TGM2 and COQ8A, though not typical members of kinome, were included in our kinase signature because of their protein kinase-like fold and reported kinase activities, respectively.^45^^,^^46^ The simplified panel contains many potential therapeutic targets, with KALRN, STK39, TGM2, PAK1 and CDK1 regulating processes and pathways that include antitumour immunity, tumourigenicity and proliferation.^47^, ^48^, ^49^, ^50^, ^51^ Notably, TGM2 and CDK1 are overexpressed in the EMT subgroup and represent potential targets for overcoming chemoresistance (Figure S3C).

### Predictive value of a simplified 11-kinase signature for stratifying chemoresponse and specific chemotherapeutic regimens

**Fig. 4:**
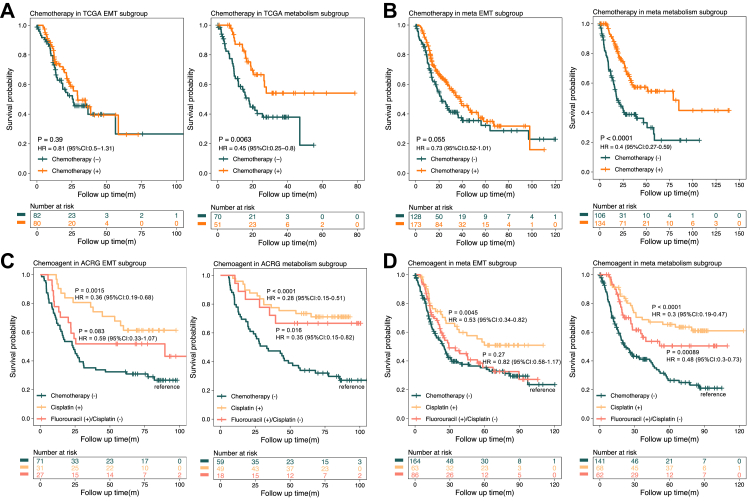
**Patient response to chemotherapy after stratification using the 11-kinase signature**. **A** Stratification of the The Cancer Genome Atlas (TCGA) cohort. The TCGA cohort was split into epithelial-mesenchymal transition (EMT, left) and metabolism (right) subgroups using the simplified 11-kinase signature. The survival difference, hazard ratios (HRs) and 95% confidence intervals (CI) between chemotherapy (+) and chemotherapy (−) in each subgroup were evaluated by univariable Cox analysis. **B** Stratification of meta-1 cohort. A combined cohort comprising the TCGA, Kosin University Gospel Hospital (KUGH), Kosin University College of Medicine (KUCM), Peking University Cancer Hospital (PUCH) and Australian cohorts was split into meta EMT (left) and meta metabolism (right) subgroups using the 11-kinase signature. The survival difference, HRs and 95% CI between chemotherapy (+) and chemotherapy (−) in each subgroup were evaluated by univariable Cox analysis. **C** Responses to cisplatin or 5-fluorouracil (5-FU) following stratification of a single cohort. The Asian Cancer Research Group (ACRG) cohort was split into EMT (left) and metabolism (right) subgroups using the 11-kinase signature. The survival difference, HRs and 95% CIs between cisplatin-based regimens, 5-FU treatment without cisplatin, and no chemotherapy in each subgroup were evaluated by univariable Cox analysis. **D** Responses in meta-2 cohort. A combined cohort comprising the TCGA, ACRG and Australian cohorts was split into meta EMT (left) and meta metabolism (right) subgroups using the 11-kinase signature. The survival difference, HRs and 95% CIs between cisplatin-based regimens, 5-FU-based treatment without cisplatin, and no chemotherapy in each subgroup were evaluated by univariable Cox analysis.

To apply more stringent evaluation and explore a wider range of clinical applications for our signature, the 11-kinase signature was subjected to further validation on additional cohorts, profiled either by RNA sequencing or gene expression array. For this, we collected patient samples from the following cohorts: TCGA, KUGH, and meta-1 (comprising the TCGA, KUGH, KUCM, PUCH, and Australian cohorts to achieve a sample size >256 in each subgroup, see Methods). For the validation, as previously described, we first subdivided the patient cohorts into two groups, EMT and metabolism based on our trained ML model with the 11-kinase signature. Within the metabolism subgroups, patients receiving chemotherapy consistently showed a significantly extended OS compared to patients who did not receive chemotherapy. The HRs for chemotherapy were 0.45 in the TCGA cohort (P = 0.0063; Fig. 4A), 0.21 in the KUGH cohort (P = 0.011; Figure S4A), and 0.4 in the meta cohort 1 (P < 0.0001; Fig. 4B). Meanwhile, as anticipated, EMT tumours identified by the signature in individual cohorts or the meta-1 cohort did not significantly benefit from chemotherapy (Fig. 4A and B, Figure S4A).

During the validation process, we observed that in the ACRG cohort, patients with EMT tumours, although not as responsive to chemotherapy as metabolism subgroup, still exhibited a noteworthy chemoresponse (P = 0.0012, HR = 0.46; Figure S4B). Since no significant difference was observed between the inhibitory effects of cisplatin in EMT and metabolism GC cell lines (GDSC database, P = 0.34, student T-test; Figure S4C and D), we hypothesised that the ACRG result might reflect the use of different chemotherapeutic agents to the previously-analysed cohorts. Therefore, the ACRG cohort was further segmented into three categories according to treatment regimens: chemotherapy (−), cisplatin (+), and 5-FU (+)/cisplatin (−) (see Methods), and survival differences were compared across these groups. In metabolism tumours, both cisplatin- and 5-FU-based treatments improved OS significantly compared to the chemotherapy (−) group, whereas in EMT tumours, only cisplatin-based treatment exhibited a significant survival benefit (Fig. 4C). This result was also obtained upon meta-2 cohort (aggregation of the TCGA, ACRG, and Australian cohorts to achieve the sample size >256 in each subgroup): cisplatin significantly enhanced survival in both subgroups, with this benefit being more pronounced in the metabolism tumours (HR_metabolism_ = 0.3 versus HR_EMT_ = 0.53). In contrast, 5-FU was effective only in metabolism tumours (P = 0.00089; HR = 0.48) (Fig. 4D). The differential impact of these two chemotherapy drugs on the survival of different GC subgroups aligned with the *in vitro* data from the GDSC (Fig. 2B, Figure S4D).

Additionally, to compare the predictive capacity of known biomarkers in chemoresponse, we subdivided the ACRG cohort based on established molecular subtyping systems of GC. Mesenchymal phenotype (MP) and epithelial phenotype (EP) represent two molecular subtypes of GC that exhibit relative resistance and sensitivity to standard chemotherapy, respectively.^21^ In addition, another prediction model utilises a transcriptome signature based on response of patient-derived xenografts to 5-FU and oxaliplatin-based chemotherapy.^20^ Using the ACRG cohort, we found that these biomarkers were not effective in stratifying patients’ response to 5-FU or cisplatin. Contrary to expectations, the subgroups predicted to be chemoresistant still demonstrated significant survival benefit post-chemotherapy, or a strong trend for this (Figure S4E and F).

Collectively, our signature demonstrated robust efficacy in identifying patients who may benefit from chemotherapy, and superior performance compared to other established molecular subtyping systems in predicting chemoresponse of specific regimens.

### The 11-kinase signature and radiotherapy response

**Fig. 5:**
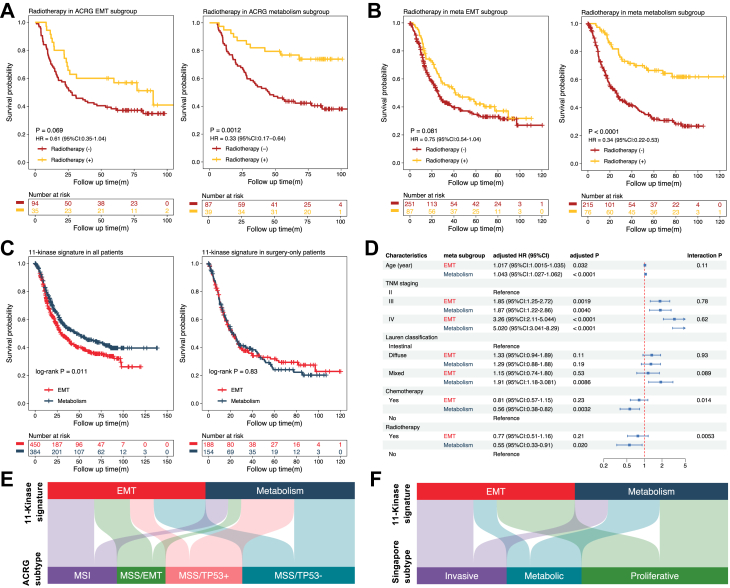
**The kinomic subgroups exhibit contrasting therapeutic response in multivariable analysis and differences to other gastric cancer subclassifications**. **A** Responses to radiotherapy following stratification of a single cohort. The Asian Cancer Research Group (ACRG) cohort was split into epithelial-mesenchymal transition (EMT, left) and metabolism (right) subgroups using the 11-kinase signature. The survival difference, hazard ratios (HRs) and 95% confidence intervals (CI) between radiotherapy (+) and radiotherapy (−) in each subgroup were evaluated by univariable Cox analysis. **B** Responses in meta-3 cohort. A combined cohort comprising the The Cancer Genome Atlas (TCGA), ACRG, University of Texas MD Anderson Cancer Center (MDACC) and Australian cohorts, was split into meta EMT (left) and meta metabolism (right) subgroups using the 11-kinase signature. The survival difference, HRs and 95% CI between radiotherapy (+) and radiotherapy (−) in each subgroup were evaluated by univariable Cox analysis. **C** Survival analysis in meta-4 cohort, comprising the ACRG, TCGA, KUGH, KUCM, PUCH, Australian, and MDACC cohorts. Survival differences between the EMT and metabolism subgroups were assessed using log-rank tests in all patients (left) and surgery-only patients (right). **D** Multivariable Cox analysis. This was undertaken on a meta cohort comprising the TCGA, ACRG and Australian cohorts. Blue bars in the forest plot denote the HRs and 95% CI of each clinical characteristic of the individual subgroup. Interaction P indicates the effect or difference of the clinical characteristic across the subgroups. **E** Comparison of 11-kinase signature-based taxonomy with ACRG subclassification, and **F** Singapore subclassification. MSI, microsatellite instability; MSS, microsatellite stability.

The efficacy of radiotherapy delivered after gastrectomy is controversial.^6^ Since effective patient stratification may help resolve this issue, we evaluated whether the 11-kinase signature predicts response to radiotherapy. Within the metabolism subgroups, radiotherapy significantly improved OS, as the HRs for radiotherapy were 0.33 in the ACRG cohort (P = 0.0012; Fig. 5A), 0.23 in the TCGA cohort (P = 0.0051; Figure S5A), and 0.34 in the meta-3 cohort (pooled ACRG, TCGA, Australian and MDACC cohorts to achieve the sample size close to 384 in each subgroup) (P < 0.0001; Fig. 5B). In contrast, patients with EMT tumours did not significantly benefit from radiotherapy across all individual cohorts and the meta-3 cohort (Fig. 5A and B, Figure S5A). Notably, the meta-3 cohort was designed to achieve a sample size of 384 per kinomic subgroup; however, the actual number averaged slightly lower at 315. As a retrospective study utilising all publicly available data, we were unable to further increase the sample size for the radioresponse analysis.

### Multivariable analysis of clinicopathological factors following patient stratification based on the 11-kinase signature

To further clarify whether these different therapeutic responses between kinomic subgroups are related to clinicopathological characteristics, we combined the ACRG, TCGA, KUGH, KUCM, PUCH, Australian, and MDACC cohorts to create a meta-4 cohort. We observed that although there were statistical differences in OS between patients with EMT and metabolism tumours (P = 0.011) in the meta-4, there was no survival difference for surgery-only patients between these two subgroups (P = 0.83; Fig. 5C, Table S4). This led us to suspect that the survival benefit for patients with metabolism tumours may derive from treatment rather than intrinsic tumour characteristics. To formally address this possibility, we interrogated the relationship between the two kinomic subgroups and other clinicopathological characteristics using this meta cohort. No significant relationships were found between the EMT and metabolism subgroups and age, sex, TNM staging, Lauren classification, tumour location, number of patients undergoing chemotherapy, and number of patients undergoing radiotherapy (Table S4). The assumptions of the Cox model were met in both EMT and metabolism subgroups. For the continuous variable, age, the Schoenfeld tests were non-significant (EMT: P = 0.79; metabolism: P = 0.52), indicating that the effect of age does not change over time. Furthermore, the linearity assumption was also supported, as likelihood-ratio tests comparing the linear age term to a more complex restricted cubic spline model were non-significant (EMT: P = 0.074; Metabolism: P = 0.12; Figure S5B).

Univariable analysis in the meta-4 indicated that in the EMT group, OS was correlated with age, TNM staging and chemotherapy. In the metabolism group, OS was associated with age, TNM staging, Lauren classification, chemotherapy, and radiotherapy (Table S4). To further characterise independent factors for each kinomic subgroup, we compiled all cohorts with complete information of radiotherapy and chemotherapy (ACRG, TCGA and Australian cohorts), and created a meta-5 cohort. The multivariable analysis revealed that age and TNM were independent prognostic factors in EMT tumours of the meta-5 cohort, while age, TNM, Lauren subtype, chemotherapy (P = 0.0032, HR = 0.56) and radiotherapy (P = 0.020, HR = 0.55) were independent prognostic factors in metabolism tumours (Fig. 5D). No significant additive interaction was detected between kinomic subtypes and clinicopathological variables (all P > 0.05; Table S4), suggesting that there were no synergistic or antagonistic effects of subtypes and variables on survival. However, the multiplicativity interaction P values of chemotherapy and radiotherapy between the two subtypes of tumour were 0.014 and 0.0053, respectively (Fig. 5D), supporting the predictive role of our signature for treatment responses. To address whether this held for patients of different ethnicity, we extracted the White population from the TCGA cohort, and by combining this population with the ACRG cohort, which we considered predominantly Asian, we included ethnicity as a covariate in the Cox regression model. This indicated that Asian patients demonstrated significantly better prognoses than White patients in the metabolism subgroup (Figure S5C). However, this observation should be interpreted cautiously, as the TCGA cohort was generally enrolled historically earlier than the ACRG cohort, and D2 radical gastrectomy is more frequently performed in Asian GC practice. Importantly, after adjusting for ethnicity, the contrasting effects of chemoradiotherapy for the two kinase-based subgroups remained evident (Figure S5C).

### Comparison of the 11-kinase signature with other gastric cancer taxonomies

To assess the novelty of our GC subclassification, we first compared the subtyping based on our signature with a range of established molecular subtyping systems including ACRG, Singapore, KUGH and TCGA. For ACRG subclusters, 67.2% (43/64) of MSI and 73.3% (33/45) of MSS/EMT tumours corresponded to the EMT subgroup, whereas 69.0% (49/71) of MSS/TP53+ tumours associated with metabolism subgroup, contributing to a highly significant statistical correlation between our kinase signature and ACRG subtyping (P < 0.0001; Chi-square test; Table S5, Fig. 5E), although no ACRG subgroup exactly matched one of our kinase-defined subgroups. Significant relationships were also detected between KUGH (P = 0.0078; Chi-square test) and Singapore (P = 0.0055; Chi-square test) subtyping^27^ and our classification with correspondence between the majority of the KUGH mesenchymal, Singapore invasive and our EMT subgroup, and Singapore metabolic and our metabolism subgroup (Figure S5D, Fig. 5F, Table S5). However, as with the ACRG comparison, the subgroups do not have a simple relationship with ours. No significant correlation was observed between the 11-kinase signature-derived and TCGA subclusters (Table S5, Figure S5E).

Regarding the clinical relevance of GC taxonomies, unlike our 11-kinase signature—where survival differences among subgroups are entirely attributable to treatment benefits (Fig. 5C, Table S4)—existing classifications show varying prognostic implications. No significant differences in OS were observed among TCGA subtypes in either overall patients or surgery-only patients (Figure S5F). In contrast, the ACRG classification revealed substantial OS differences across both overall patients and surgery-only patients, with the MSI subtype showing the most favourable prognosis and the MSS/EMT subtype the poorest (Figure S5G). According to multivariable analysis, the TCGA classification did not effectively stratify patients by chemotherapy response, but its GS subtype may benefit from radiotherapy (P = 0.031; Figure S5H). The ACRG classification did not clearly stratify patients by chemo or radiotherapy response, and its MSI subtype may not benefit from chemotherapy (P = 0.57; Figure S5I). Consequently, neither classification system showed a significant subtype-related chemotherapy or radiotherapy interaction, suggesting limited utility in predicting treatment response.

### The molecular landscape of EMT and metabolism subgroup tumours

**Fig. 6:**
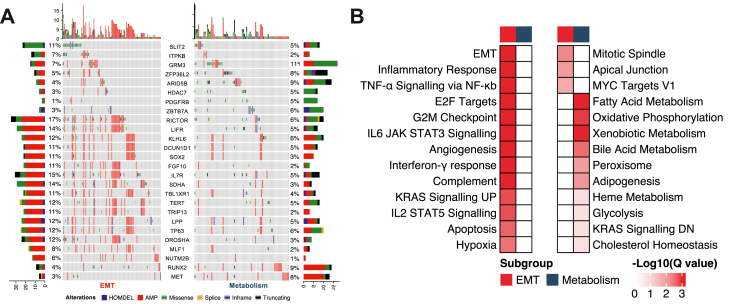
**Molecular landscapes of the EMT and metabolism subgroups**. **A** OncoPlot summary of significantly mutated, amplified or deleted oncogenes between kinomic subgroups in The Cancer Genome Atlas (TCGA) cohort. The histogram on top shows the number of genetic alterations among these oncogenes that were observed for each corresponding case. The bars on the side show the number of genetic alterations that were observed in individual oncogenes in the epithelial-mesenchymal transition (EMT) or metabolism subgroups. **B** Enriched hallmark gene sets at the transcription level between the two subgroups of the Asian Cancer Research Group (ACRG) cohort. HOMDEL, homozygous deletion; AMP, amplification; DN, down.

To understand the molecular mechanisms underlying these differences in therapeutic response, we first interrogated genomic alterations. Despite the absence of a statistically significant difference in tumour mutation burden across the kinomic subgroups (P = 0.41; Student's t-test; Figure S6A), a notable distinction was observed in the pattern of genomic alterations, particularly amplifications across individual genes (Table S6). Overall, 26 oncogenes were differentially altered between the kinomic subgroups, with a prevalence of gene amplification events for these oncogenes in EMT tumours (Fig. 6A). Gene ontology analysis across these 26 oncogenes revealed an enrichment in biological processes including cell cycle regulation, stem cell characteristics and apoptosis (Figure S6B). This approach also highlighted potential targeted therapy opportunities for the EMT subgroup, such as amplification of the mTOR component *RICTOR*.^52^ GSEA was also applied to determine the association of specific biological functions with particular subgroups in the ACRG cohort, highlighting that at the mRNA level, EMT tumours were significantly enriched in EMT, hypoxia, and several immune response-related processes, whereas metabolism tumours were characterised by the enrichment of metabolic pathways (Fig. 6B). Similar pathway enrichments were also observed at the proteomic level in the PUCH cohort (Figure S6C).

### Assignment of potential therapeutic targets for EMT and metabolism tumours

**Fig. 7:**
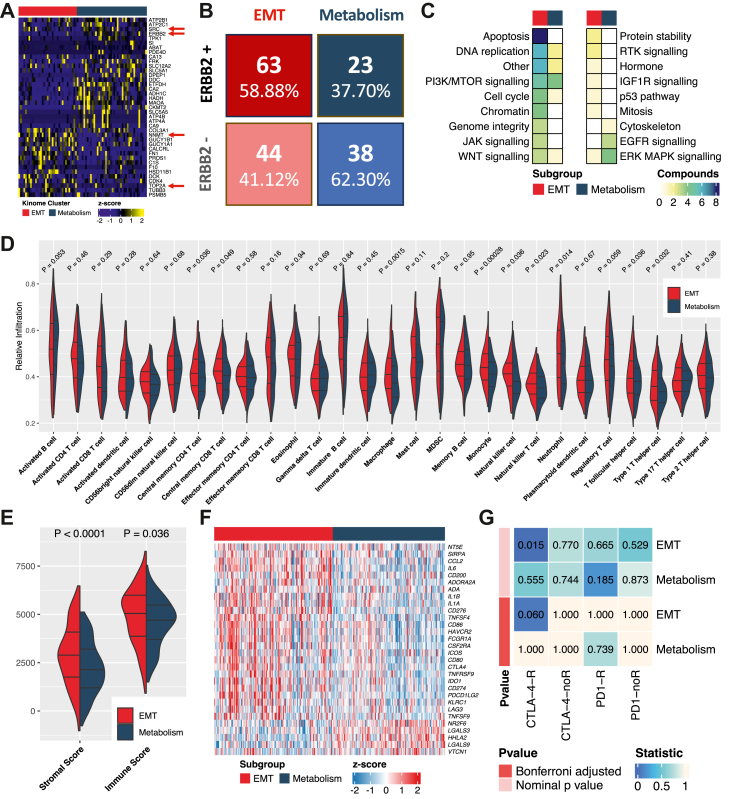
**Predicted differential sensitivity to immunotherapy and targeted therapy in the EMT and metabolism subgroups**. **A** Expression of drug targets. The heatmap highlights differentially expressed targets of FDA-approved drugs in the epithelial-mesenchymal transition (EMT) and metabolism subgroups of the Peking University Cancer Hospital (PUCH) cohort. Only the drug targets (proteins) with a log 2 fold change of abundance >1 and an adjusted P value < 0.05 between the kinomic subgroups are included. Heatmaps show drug targets (rows) arranged by hierarchical clustering and normalised protein abundance for each target. Yellow represents high protein abundance whereas navy blue represents low abundance. Subclassification of the PUCH cohort is shown at the top. The red arrows highlight the genes mentioned in the main text. **B** ERBB2 status in the kinomic subgroups of the Fudan cohort. ERBB2 status was assessed in the original study using a combination of IHC and fluorescence *in situ* hybridisation. **C** Pathway targets in the kinomic subgroups. The heatmap is colour-coded according to the number of predicted compounds that affect each listed process or pathway. **D** Tumour-infiltrating lymphocytes in the kinomic subgroups of the Asian Cancer Research Group (ACRG) cohort. **E** Predicted immune score and stromal score for the kinomic subgroups of the ACRG cohort. **F** Expression of immune receptor–ligand pairs and immunotherapy targets in the kinomic subgroups of the ACRG cohort. Only the immune molecules (genes) with a log2 fold change of mRNA expression >0.2 and an adjusted P < 0.05 between the kinomic subgroups are included. **G** Response prediction for PD-1 and CTLA-4 therapy in the kinomic subgroups of the ACRG cohort. The heatmap shows the significance of gene expression association between the kinomic subgroups of ACRG cohort and a cohort of melanoma patients with different immunotherapy outcomes.

Given the distinct molecular features observed for the EMT and metabolism subgroups, we mined FDA-approved drug targets from the Human Protein Atlas to identify subgroup-selective therapeutic targets. For instance, the pronounced expression of NNMT might explain the resistance to chemoradiotherapy in the EMT subgroup.^53^ In addition, TOP2A represents a potential therapeutic target in the chemoresistant EMT tumour subtype^54^ and SRC represents an ‘actionable’ target for metabolism tumours (Fig. 7A). In bulk protein data, ERBB2 trended higher expression in the metabolism subgroup in two cohorts (Fig. 7A, Figure S7A). However, pathological assessment in the Fudan cohort^24^ revealed a higher proportion of membrane-localised ERBB2 expression and ERBB2 amplification in the EMT subgroup (P = 0.013, Chi-square test; Fig. 7B). We then interrogated patient responses to anti-ERBB2 therapy in the Fudan cohort. However, it should be noted that this cohort differs markedly from others used in our study. The Fudan cohort predominantly comprised patients with advanced-stage disease (78.1% stage III–IV versus 56.8% on average in the other cohorts), received neoadjuvant rather than adjuvant chemotherapy, and underwent fewer treatment cycles (65.0% < four cycles). In addition, the surgical resection rate was markedly lower (41.25% versus 100% in the other cohorts). In the EMT subgroup, neoadjuvant chemotherapy plus anti-ERBB2 treatment resulted in a higher response rate than chemotherapy alone (64.3% versus 40.0%, P = 0.036, Chi-square test; Table S7). No such difference was observed in the metabolism subgroup (40.0% versus 48.8%, P = 0.56, Chi-square test; Table S7), suggesting that anti-ERBB2 therapies might be applicable to this tumour subtype under current clinical guidelines.^8^^,^^12^ However, no OS difference was observed for either the EMT or metabolism groups when chemotherapy plus anti-ERBB2 treatment was compared to chemotherapy alone (Figure S7B).

To further mine for subgroup-selective drugs based on the gene expression profile, we predicted IC50 values for specific drugs using ACRG gene expression data and regression models fitted with the expression profiles of GDSC cell lines and their drug IC50 matrix.^43^ This predicted that although there were more selective drugs for the EMT subgroup, the drugs included in the current mainstream chemotherapy (5-FU and oxaliplatin) and targeted therapy regimens (lapatinib and afatinib) for GC,^4^^,^^8^^,^^11^^,^^12^^,^^55^^,^^56^ were more effective against metabolism tumours (Figure S7C and D). This is consistent with patients with metabolism tumours exhibiting a better prognosis after treatment compared to those with EMT tumours (Fig. 5C, Table S4). Rapamycin (mTOR inhibitor) and Trametinib (MEK1/2 inhibitor) exhibited predicted enhanced efficacy for EMT and metabolism tumours, respectively, consistent with our *in vitro* findings.^16^ When mapping the compounds to their target pathways, the predicted subgroup-specific drugs primarily exerted effects on EMT tumours through apoptosis or DNA replication pathways, while specific drugs in metabolism tumours mainly acted on the EGFR and ERK pathways (Fig. 7C).

We also investigated the correlation of subgroups with immune-related gene expression signatures.^15^^,^^57^ A significantly higher abundance of stromal scores, immune scores, as well as immune cell populations, including macrophage, monocyte, natural killer cells, and T helper cells, were observed in EMT tumours, while activated B cells were more prevalent in metabolism tumours (Fig. 7D and E). Additionally, higher expression of 25 immune receptor-ligand or immune checkpoint genes were identified in EMT tumours (Fig. 7F). In the Australian cohort,^26^ further IHC analysis indicated a trend toward a higher frequency of PD-L1–positive cases (CPS >1) in EMT tumours relative to metabolism tumours (19.51% versus 6.25%, P = 0.17; Fisher's exact test; Figure S8A and B). A similar trend was also observed in the Fudan cohort at bulk proteomic level, where 5/116 patients with EMT tumours exhibited PD-L1 expression, compared to none among the 64 patients with metabolism tumour (4.13% versus 0%, P = 0.166; Fisher's exact test; Figure S8C). The SubMap analysis was utilised to find potential immunotherapy benefit for kinomic subgroups using the available data of clinical response and gene expression from a melanoma immunotherapy dataset,^58^ suggesting that the EMT subgroup in the ACRG cohort shared high similarity with the anti-CTLA4 response group in the melanoma cohort (P = 0.015, adj.P = 0.060), while no significant correlation between the metabolism subgroup and immunoresponse in melanoma was observed. However, neither kinomic subgroup had a significant similarity with the anti-PD1 response group of the melanoma dataset (Fig. 7G).

## Discussion

In this study, using a RF approach, a ML model was trained using cell line–derived genomic data and validated across seven independent patient cohorts encompassing both Asian and Western populations. The resulting classifier, representing an 11-kinase gene expression signature, could be applied to treatment-naïve tumour samples to help clinicians identify patients likely to benefit from conventional chemoradiotherapy. Clinically, GC assigned into a ‘metabolism’ subgroup exhibited improved OS after chemotherapy or radiotherapy compared with patients who did not receive these treatments, with a mean absolute increase in 5-year OS of above 30%. In contrast, patients with EMT tumour did not benefit from chemotherapy or radiotherapy. In addition, the kinase signature itself and contrasting distribution of immuno- and other small molecule targets between the two subgroups may inform strategies to overcome chemo- and radioresistance and future directions for precision therapy of GC. Integration of the signature with established or emerging predictive biomarkers (e.g., ERBB2, PD-L1) may identify alternative treatments for the EMT subgroup and hence improve personalised treatment of GC, although it will be important to confirm the sensitivity of this subgroup to such treatments using appropriate patient cohorts.

Metabolic activities and EMT processes have been confirmed by multiple studies to vary between chemotherapy-sensitive and insensitive GC, leading to identification of potential predictive biomarkers.^20^^,^^21^^,^^27^ However, different from other RNA biomarkers only focused on chemosensitivity, the 11-kinase signature is validated across mRNA and protein levels to predict benefits of chemotherapy as well as radiotherapy in GC. It also outperforms previously-identified strategies, including in-use pathology subclassification and other molecular subtypes, in predictive accuracy and a broader applicability to patient populations.^13^^,^^20^, ^21^, ^22^^,^^27^ Furthermore, it is a relatively simple signature amenable to further clinical evaluation and ultimately application.

TGM2 and CDK1, two feature kinases identified in the final model and highly expressed in the EMT subgroup, have been implicated in tumour progression, EMT, metastasis, and chemoresistance.^47^^,^^59^, ^60^, ^61^ In breast cancer, TGM2 activates NF-κB, which in turn elevates HIF-1α and EMT transcription factors (ZEB1/2, Snail, Twist) via a non-canonical NF-κB/HIF-1α pathway, contributing to treatment resistance.^59^ CDK1 mediates crosstalk between the NF-κB and β-catenin pathways in response to *Helicobacter pylori* infection, and its silencing sensitises GC to chemotherapy.^60^^,^^61^ These finding align with our gene set enrichment analyses, revealing up-regulated EMT and hypoxia pathways may be involved in mediating drug resistance to 5-FU (Fig. 6B, Figure S6C). Hypoxia can impair DNA replication and repair via thymidylate synthase inhibition, leading to 5-FU resistance.^62^ HIF-1α may further enhance EMT through activation of Hedgehog and TGF-β.^63^ Previous studies suggested that TGF-β induced EMT plays a role in the development of 5-FU resistance in GC cell lines, while EMT suppression can restore the drug sensitivity.^64^^,^^65^ Consistent with these *in vitro* data, the patients in the ACRG MSS/EMT subtype were mainly assigned into the 5-FU resistant subgroup (Table S5). In the metabolism subgroup, several cellular pathways associated with 5-FU sensitivity were identified, such as the p53 pathway, which mediates cell cycle arrest and apoptosis in GC cells with functional p53^66^ and particular metabolic processes (Fig. 6B, Figure S6C). Consistent with aberrant mitochondrial metabolism being associated with chemoresistance, mitochondrial function was significantly downregulated in 5-FU resistant Hela cells,^67^ and we observed down-regulated oxidative phosphorylation in EMT GC (Fig. 6B).

A positive correlation has been observed between T cell infiltration and the expression of EMT-related genes in many types of cancer, with the suggestion that EMT tumours are more likely to benefit from immunotherapy.^68^^,^^69^ Interestingly, KALRN, another key kinase in our model and downregulated in the EMT subgroup, has been associated with modulation of immune sensitivity. Loss or mutation of KALRN enhances antitumour immunity and response to immunotherapy, serving as a potential biomarker for patient stratification in cancer immunotherapy.^51^ Correspondingly, the EMT tumours in the ACRG cohort displayed high immune infiltration and immune gene expression. Furthermore, submap analysis indicated that anti-CTLA-4 therapy may hold potential value in the ACRG EMT subgroup (Fig. 7F). Based on these analyses, immunotherapy appears to be a potential treatment option for patients in the EMT subgroup that do not benefit from current chemotherapy and radiotherapy. Interestingly, immune-related pathways demonstrated completely opposite enrichment results at the cellular and patient level, with immune pathways being significantly enriched in the metabolism GC cell lines,^16^ while they were highly activated in the EMT subgroup of the ACRG cohort (Fig. 6B). This likely reflects differences between *in vitro* and *in vivo* conditions, including the presence of a complex tumour microenvironment in the latter.

Our study's main limitation is the use of retrospective publicly-available patient datasets, which were generated without a prespecified protocol and randomisation design. Although the potential for bias in such a study design was mitigated by adherence to the TRIPOD guidelines^23^ and the use of a ‘hard’ endpoint of death, limitations remain due to incomplete clinical data and heterogeneous treatment protocols across cohorts. Censoring rates of actual data in this study varied substantially across cohorts (16.07%–73.17%), though reasons for censoring were not reported. Higher censoring rates may be attributed to shorter follow-up periods. For instance, the PUCH cohort had the shortest study period (<5 years), which could partially explain its highest censoring rate (73.17%). Nonetheless, several factors mitigate bias in survival estimates from high censoring rates. First, given the median OS of GC (∼16 months^70^) and the median follow-up in our study exceeding 23 months, most patients had sufficient time for the event (death) to occur, minimising the likelihood that censoring affected the robustness of our analyses. Second, the high censoring rate was primarily attributable to patients who remained alive at the end of study rather than loss to follow-up or early withdrawal; such administrative censoring is non-informative and unlikely to bias survival estimates. Overall, these findings and limitations warrant validation in future prospective studies with larger sample sizes, standardised treatment protocols, and extended follow-up periods.

In a non-randomised context, HRs are conditional on survival and may be affected by time-dependent selection bias.^71^ Patients who receive additional therapy often have better physical or socioeconomic status, leading to more favourable prognoses and biased treatment effect estimates through the “depletion of susceptible” phenomenon. This issue is compounded by unmeasured confounders—such as dose intensity, subsequent therapies, and comorbidities—which could not be adjusted for in our model. Using kinases as molecular predictors simplifies the underlying biological complexity. While prior work supports their utility in stratifying GC cell lines, this approach may limit predictive performance on tissues, especially when feature sets are compressed.^16^ Incorporating non-kinase features could improve accuracy by better capturing tumour heterogeneity. Together, these sources of bias may also contribute to model misspecification, limiting the validity of the HRs for the kinase signature and treatment effects.

Like most tumour subtyping studies, this kinase signature relies on data normalised across samples, which reduces technical noise, allowing for more accurate classification of tumour subtypes and identification of differential molecules and pathways.^15^^,^^21^^,^^25^^,^^27^ However, this approach poses challenges for clinical implementation due to difficulties in obtaining scaled values for individual patients in isolation. One potential solution is to normalise the data within each sample across variables (genes or proteins), rather than across all samples. This approach assumes that the relative expression levels between genes or proteins are more critical than their absolute values. For instance, the ratio of Bcl-2 to Bax can determine whether a cell survives or undergoes apoptosis.^72^ By normalising data in this manner for the model training, we would be able to quantify and standardise the data for individual patients, facilitating the prediction of classifications (EMT or Metabolism) based on the normalised data.

Although our study primarily focuses on model development and validation, we have aligned it with key principles from the Medical Algorithmic Audit^73^ and STANDING Together^74^ frameworks by emphasising dataset representativeness, transparency of data sources, and the assessment of potential algorithmic bias. Specifically, we evaluated multiple ML algorithms and carefully partitioned datasets to ensure balanced and unbiased model construction. The final model consistently stratified patients according to treatment responses across cohorts from diverse ethnic and regional backgrounds (Asian and Western), supporting the generalisability and fairness of the signature. Going forward, we plan to incorporate formal auditing procedures during prospective deployment. Since the model inputs are based on RNA expression, we are standardising data acquisition and quantification protocols, and we plan to conduct a prospective clinical study using a RT–PCR assay to validate the kinase signature. This assay can be integrated into the routine surgical–pathological workflow, adding a 2-h molecular diagnostic step to assist clinicians and patients in therapeutic decision-making.

### Conclusions

By training a ML model on a broad GC cell line panel, then cross-validating the model across publicly-accessible datasets, we developed and validated a 11-kinase signature which can predict benefit from chemotherapy and radiotherapy in heterogeneous cohorts of patients with stage II-IV GC. We also utilise the signature to derive a subclassification of GC with subtype-specific molecular features and therapeutic targets, highlighting the potential application of this translational research for survival prediction and therapeutic guidance.

## Contributors

C. H. conducted the prediction works, analysed the results, and wrote the first draft. S. S., Y. W. and P. L. developed and revised python script. C. C., R. A. B. and Y. W. revised cohort data. J. S., T. K., L. K. N. and X. S. were responsible for the methodology and analysis design. C. A. M. conducted the PD-L1 IHC analysis on the Australian cohort. H. S. reviewed all statistical analysis. J. S., T. K. and R. J. D. supervised this study. L. K. N., J. S., S. S., A. B., X. S. and R. J. D. revised the manuscript. A. B. and R. A. B. can provide access to the Australian cohort. C. H. and R. J. D. can provide access to all the processed data. C. H. and S. S. have verified the underlying data. R. J. D. conceived and designed the study. All authors read and approved the final manuscript.

## Data sharing statement

All data supporting the findings of this study are available from public repositories or in the Supplementary Materials. The in-house cell line proteomic data are available via the ProteomeXchange Consortium (PXD048538). The PUCH and Fudan datasets are deposited under accession numbers PXD008840 and PXD024255 (via iProX, Project ID IPX0002116000; http://www.iprox.cn/), respectively. TCGA gastric cancer data are accessible through the TCGA portal (http://cancergenome.nih.gov) as TCGA-STAD RNA-seq level 3 data. Additional genomic datasets are available from the NCBI Gene Expression Omnibus under accession numbers GSE26899 (KUGH), GSE26901 (KUCM), GSE28541 (MDACC), GSE51105 (Australian), GSE15460 (Singapore), and GSE66229 (ACRG).

The training and validation datasets, along with prediction models and python script, are deposited at https://zenodo.org/records/13912368.

Requests for access to the Australian cohort should be directed to Alex Boussioutas (alexb@alfred.org.au). Requests regarding other datasets or analysis code should be directed to Roger J. Daly (roger.daly@monash.edu).

## Declaration of interests

The authors declare that they have no competing interests.

## Appendix A Supplementary data

Table S6

Reagent Validation File
