# Supplementary material for: Identification and validation of an 11-kinase signature that predicts chemo- and radiosensitivity in gastric cancer
Source: eBioMedicine. 2026 Feb 11;125:106154. doi: 10.1016/j.ebiom.2026.106154 (PMC12914827; doi:10.1016/j.ebiom.2026.106154)
Supplement: Supplementary Materials [file mmc1.pdf]

Supplementary Materials for

Identification and validation of an 11-kinase signature that predicts chemo- and radiosensitivity in gastric cancer

Changyuan Hu, Sung-Young Shin, Yanan Wang, Chenbin Chen, Pei Liu, Rita A. Busuttil, Yunjian Wu, Catriona A. McLean, Hongying Shi, Terry Kwok, Lan K. Nguyen, Jiangning Song, Alex Boussioutas, Xian Shen, Roger J. Daly

*Corresponding author: Roger J. Daly, roger.daly@monash.edu

This PDF file includes:

Figures S1 to S8
Tables S1, S3 to S5, S7

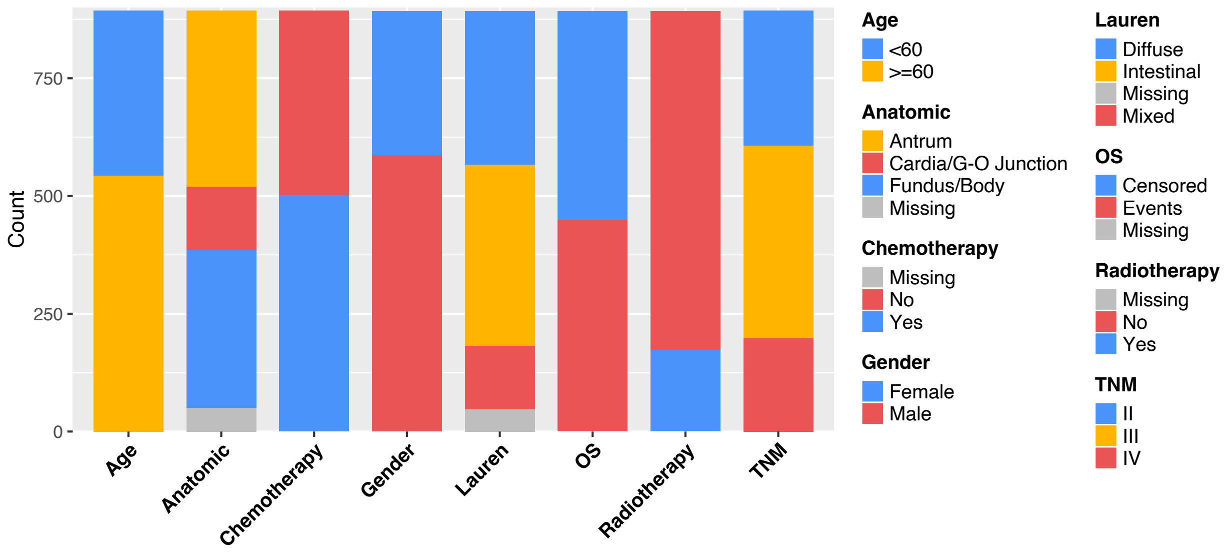

Figure S1. Characteristics of the cohorts and profiling datasets

Patient demographic and clinical characteristics overview. The x-axis represents different categories of clinical characteristics. The y-axis indicates the counts of cases in each category. The colours within the bars correspond to subcategories as shown.

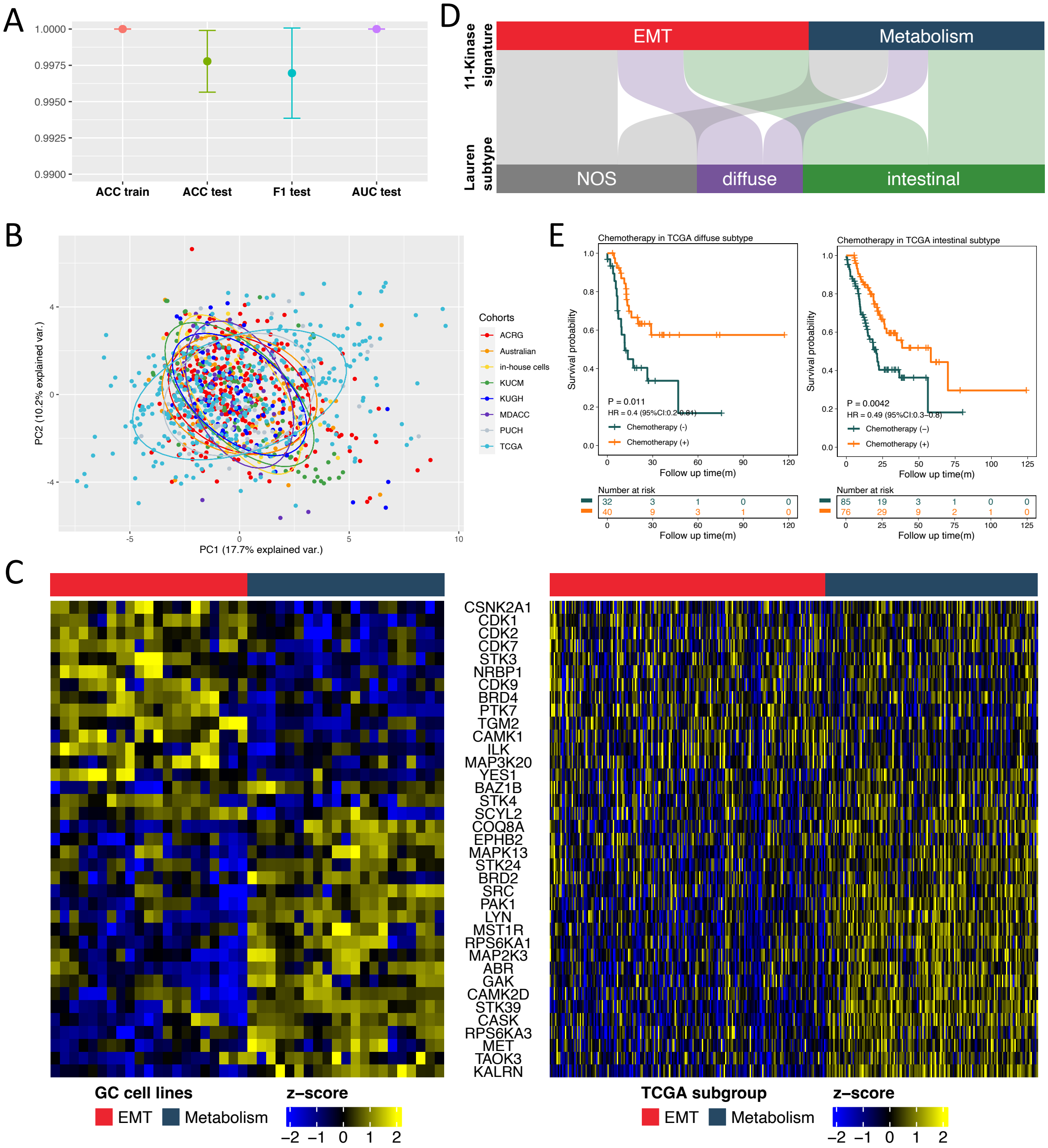

Figure S2 Development of a 37-kinase signature and its comparison with the Lauren subclassification
A Prediction accuracy of 37-kinase signature in the internal training and test cohorts based on cell line proteomics data. Data are presented as mean ± 95% confidence intervals. **B** Principal component analysis of various cohorts. The patients from eight cohorts distributed along the first two principal components by their individual profiling data, with PC1 explaining 17.7% of the variance and PC2 explaining 10.2%. **C** Comparison of the expression pattern of 37 kinases between EMT and metabolism subgroups from GC cell proteome data (left) and TCGA RNA sequencing data (right). Heatmaps represent protein abundance (left) and RNA expression level (right) of 37 kinases from two datasets. Yellow represents high expression whereas navy blue represents low expression level. Subclassification of the cell lines or patients are shown at the top. **D** Comparison of the 37-kinase signature with Lauren subclassification in the TCGA cohort. **E** Chemosensitivity of Lauren subtypes. The TCGA cohort was split into diffuse (left) and intestinal (right) subtypes by Lauren subclassification. The survival difference, HRs and 95% CI between chemotherapy (+) and chemotherapy (-) in each subgroup were evaluated by univariate Cox proportional hazards regression analysis. ACC, accuracy; AUC, area under the ROC curve; NOS, not otherwise specified.

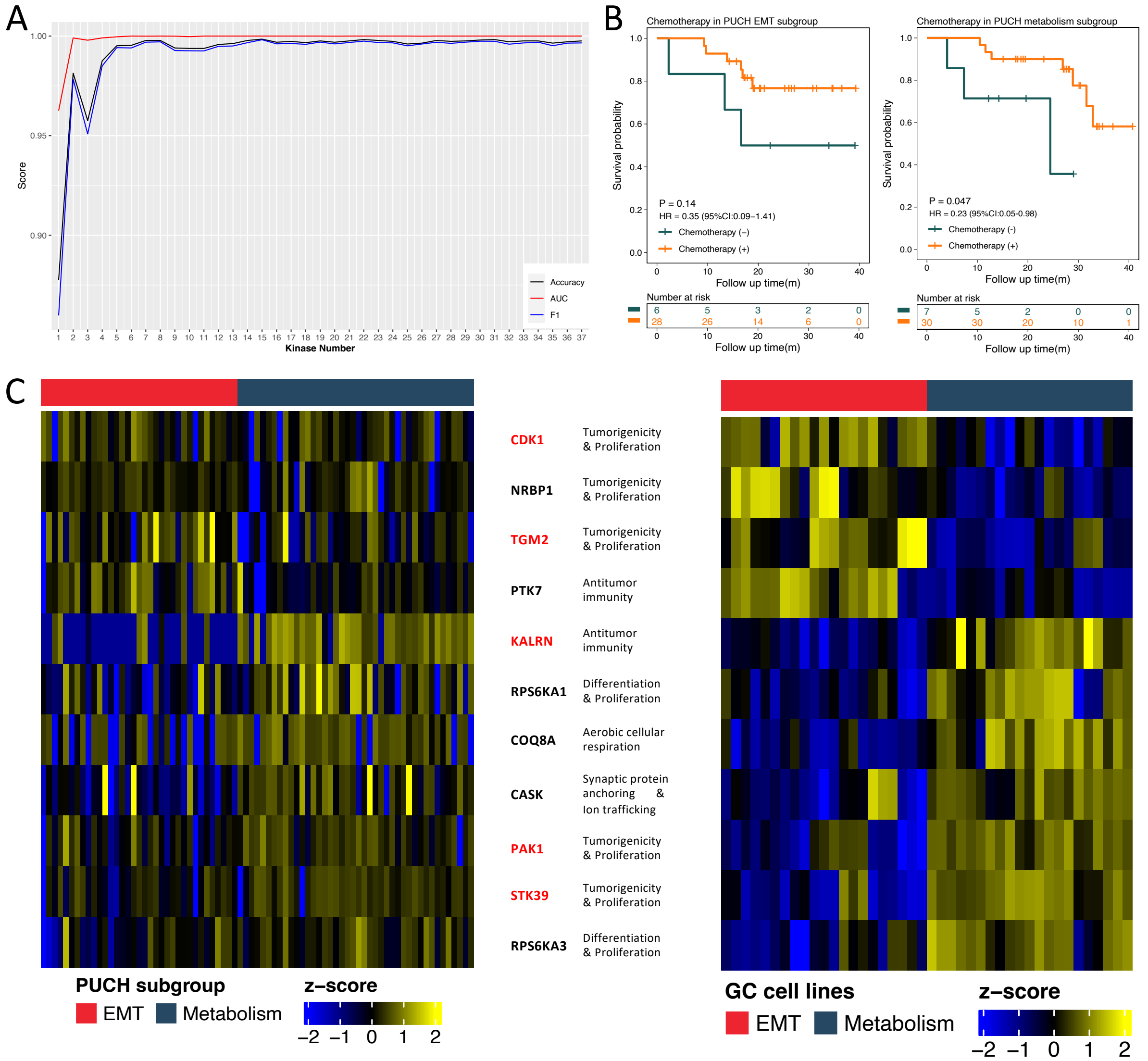

Figure S3 Simplification and validation of the kinase signature

A Performance of 37 predictive models with different number of input kinases. The model numbers, corresponding to the number of kinases used in model construction, were arranged in ascending order along the x-axis. The y-axis represents the accuracy, F1 score and AUC of each model. **B** Stratification of the PUCH cohort using the simplified 11-kinase signature. The cohort was subdivided into EMT (left) and metabolism (right) subgroups. The survival difference, HRs, 95% CIs between chemotherapy (+) and chemotherapy (-) in each subgroup were evaluated by univariate Cox proportional hazards regression analysis. **C** The expression pattern of 11 kinases between EMT and metabolism subgroups post-stratification: the PUCH cohort (left panel), cell line panel (right panel). Heatmap represents protein abundance of 11 kinases. Yellow represents high expression whereas navy blue represents low expression level. Subclassification of the cell lines or patients are shown at the top. The biological functions of 11 kinases are noted on the right, and actionable kinases are marked as red.

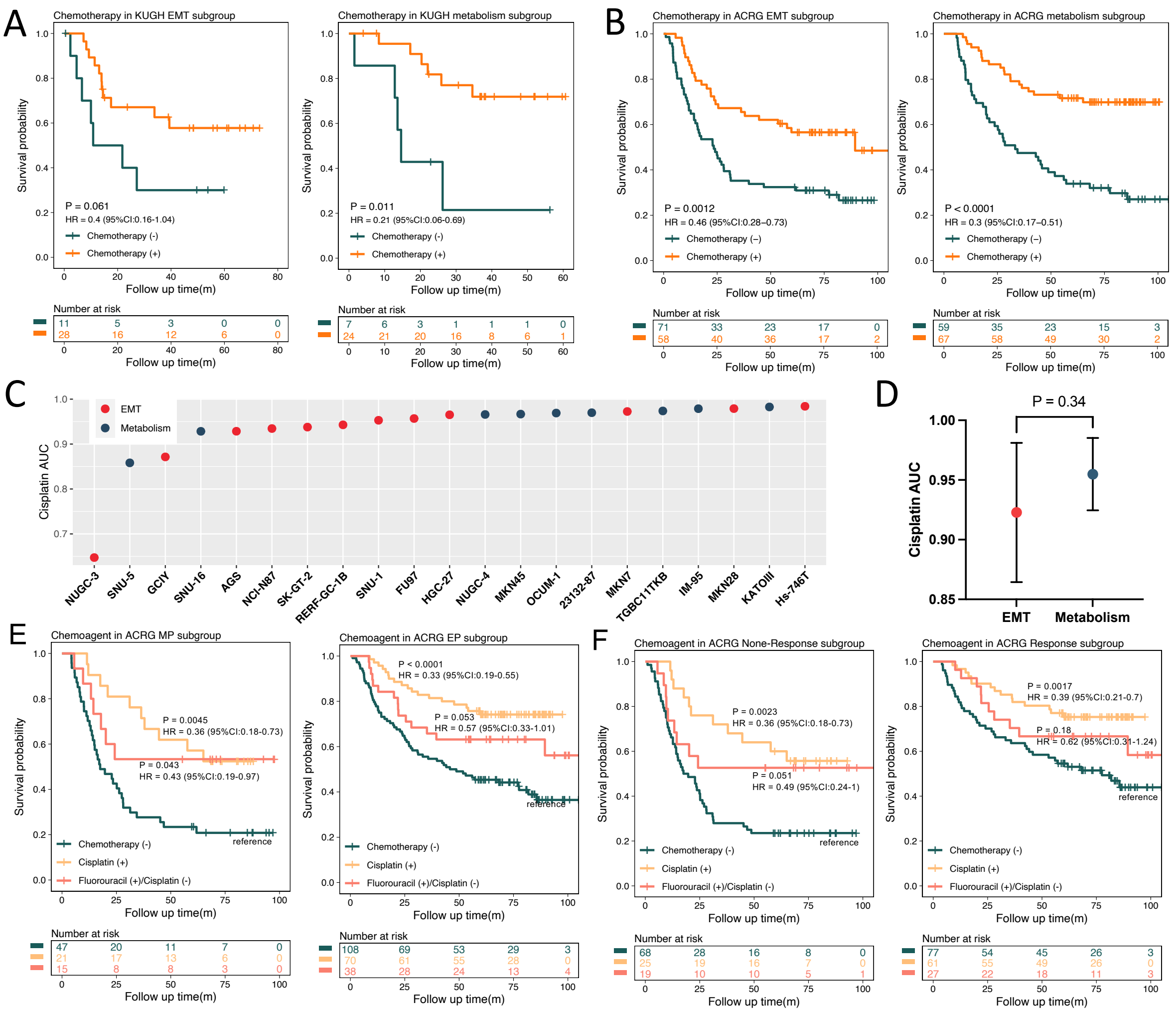

Figure S4 Effects of chemotherapy after patient stratification by 11-kinase signature and other published biomarkers

A Stratification of the KUGH cohort. This was split into EMT (left) and metabolism (right) subgroups using the 11-kinase signature. The survival difference, HRs, 95% CIs between chemotherapy (+) and chemotherapy (-) in each subgroup were evaluated by univariate Cox proportional hazards regression analysis. **B** Stratification of the ACRG cohort using the 11-kinase signature. The survival difference, HRs and 95% CI between chemotherapy (+) and chemotherapy (-) for the EMT (left) and metabolism (right) subgroups were evaluated by univariate Cox proportional hazards regression analysis. **C** Cisplatin sensitivity of GC cell lines in the Cancer Cell Line Encyclopedia. EMT and metabolism GC cell lines are indicated and exhibit a significant difference in drug sensitivity, as measured by the area under the curve (AUC). **D** A statistical analysis of cisplatin sensitivity between EMT-like and metabolism-like GC cell lines (student T-test). Data are presented as mean ± 95% CI. **E** Stratification of the ACRG cohort into MP (mesenchymal phenotype, left) and EP (epithelial phenotype, right) subgroups according to Oh *et al.*[1]. The survival difference, HRs and 95% CI between cisplatin-based regimens, fluorouracil-based treatment without cisplatin, and chemotherapy (-) in each subgroup were evaluated by univariate Cox proportional hazards regression analysis. **F** The ACRG cohort was split into no-response-like (left) and chemo-response-like (right) subgroups according to Na *et al.*[2]. The survival difference, HRs and 95% CI between cisplatin-based regimens, fluorouracil-based treatment without cisplatin, and chemotherapy (-) in each subgroup were evaluated by univariate Cox proportional hazards regression analysis.

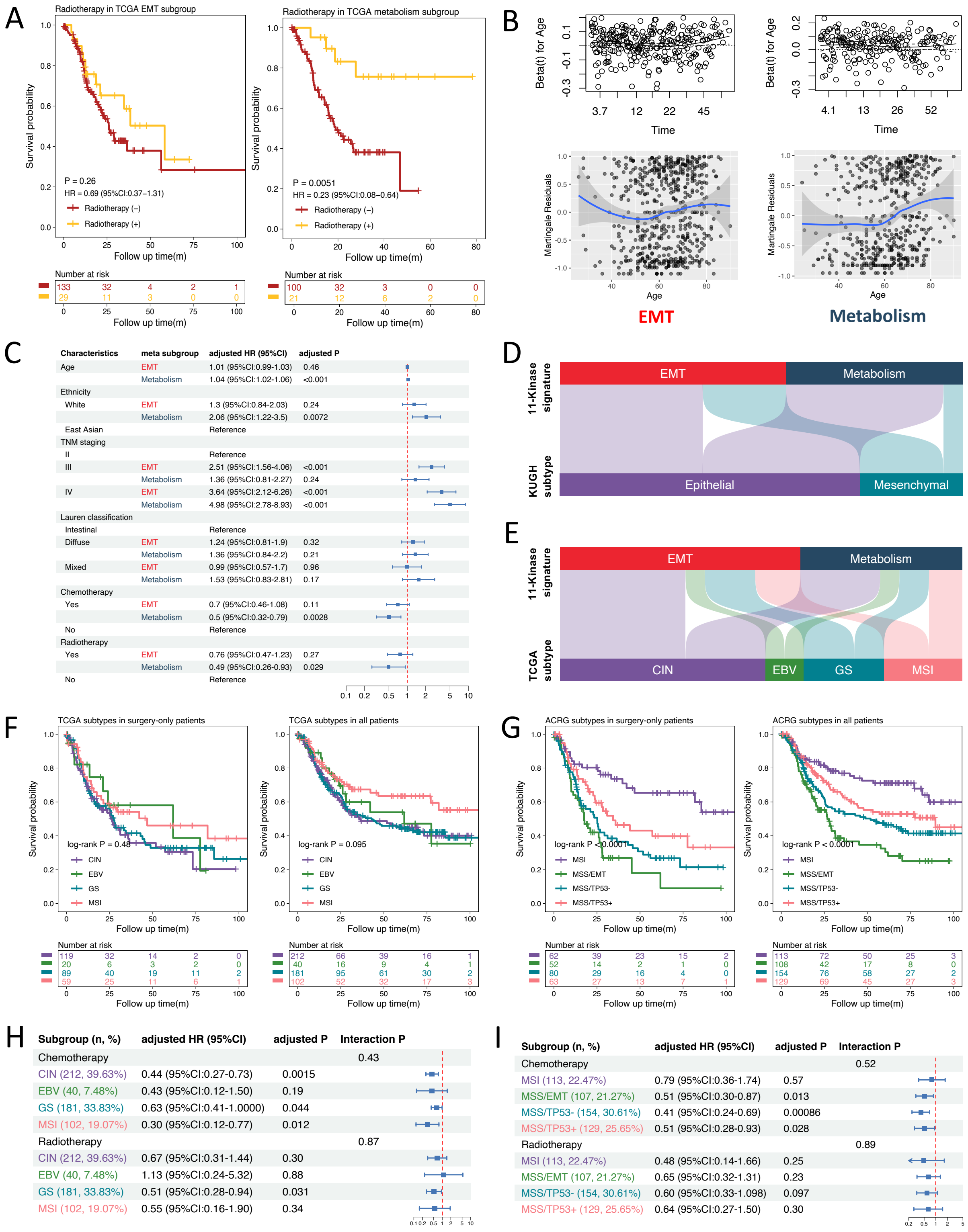

Figure S5 Comparison of radiosensitivity in kinomic subgroups stratified by 11-kinase signature and corresponding taxonomy with other gastric cancer subclassifications

A Stratification of the TCGA cohort. This was split into EMT (left) and metabolism (right) subgroups using the 11-kinase signature. The survival difference, HRs and 95% CI between radiotherapy (+) and radiotherapy (-) in each

subgroup were evaluated by univariate Cox proportional hazards regression analysis. **B** Assessment of proportional hazards and linearity assumptions for age. Top panels: Schoenfeld residuals plotted against time for the EMT (left) and metabolism (right) subgroups, with smoothed curves and 95% confidence bands. The smoothed curves are approximately flat and centred around zero, showing no clear time-dependent trends, suggesting that the proportional hazards assumption for age is satisfied. Bottom panels: Martingale residuals plotted against age show random scatter around zero, indicating that the linearity assumption for age in the Cox model is reasonably met. **C** Multivariable Cox analysis. This was undertaken on a meta cohort comprising White patients from the TCGA cohort and all patients from the ACRG cohort. Blue bars in the forest plot denote the HRs and 95% CI of each clinical characteristic of the individual subgroup. **D** Comparison of the 11-kinase signature with KUGH subclassification, and **E** TCGA subclassification. **F** Survival analysis in a merged ACRG and TCGA cohort. Survival differences between the TCGA subgroups were assessed using log-rank tests in surgery-only patients (left) and all patients (right). **G** Survival analysis in a merged ACRG and TCGA cohort. Survival differences between the ACRG subgroups were assessed using log-rank tests in surgery-only patients (left) and all patients (right). **H** Multivariable Cox analysis. This was undertaken on a merged ACRG and TCGA cohort. Blue bars in the forest plot denote the HRs and 95% CI of each clinical characteristic of the individual subgroup. Interaction P indicates the effect or difference of the clinical characteristic across the TCGA subgroups. **I** Multivariable Cox analysis. This was undertaken on a merged ACRG and TCGA cohort. Blue bars in the forest plot denote the HRs and 95% CI of each clinical characteristic of the individual subgroup. Interaction P indicates the effect or difference of the clinical characteristic across the ACRG subgroups.

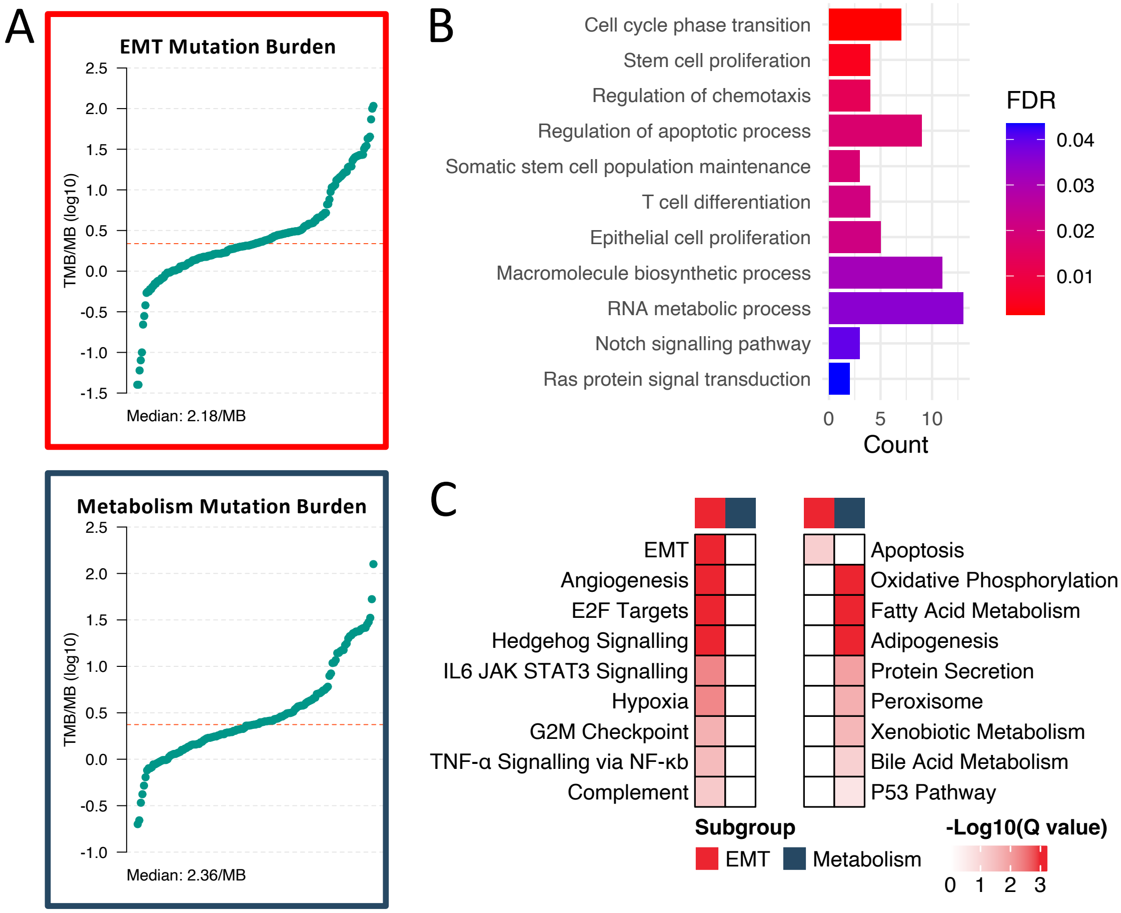

Figure S6 Molecular features of the EMT and metabolism subgroups

A Comparison of tumour somatic mutations per MB (TMB) between EMT (top) and metabolism (bottom) subgroup of TCGA cohort. **B** Enriched biological processes across 26 differentially altered oncogenes. Each bar represents the count of oncogenes associated with the respective biological process. The significance of the enrichment is coloured by gradient from red (FDR low) to blue (FDR high). **C** Enriched hallmark gene sets at the proteome level between kinomic subgroups of the PUCH cohort.

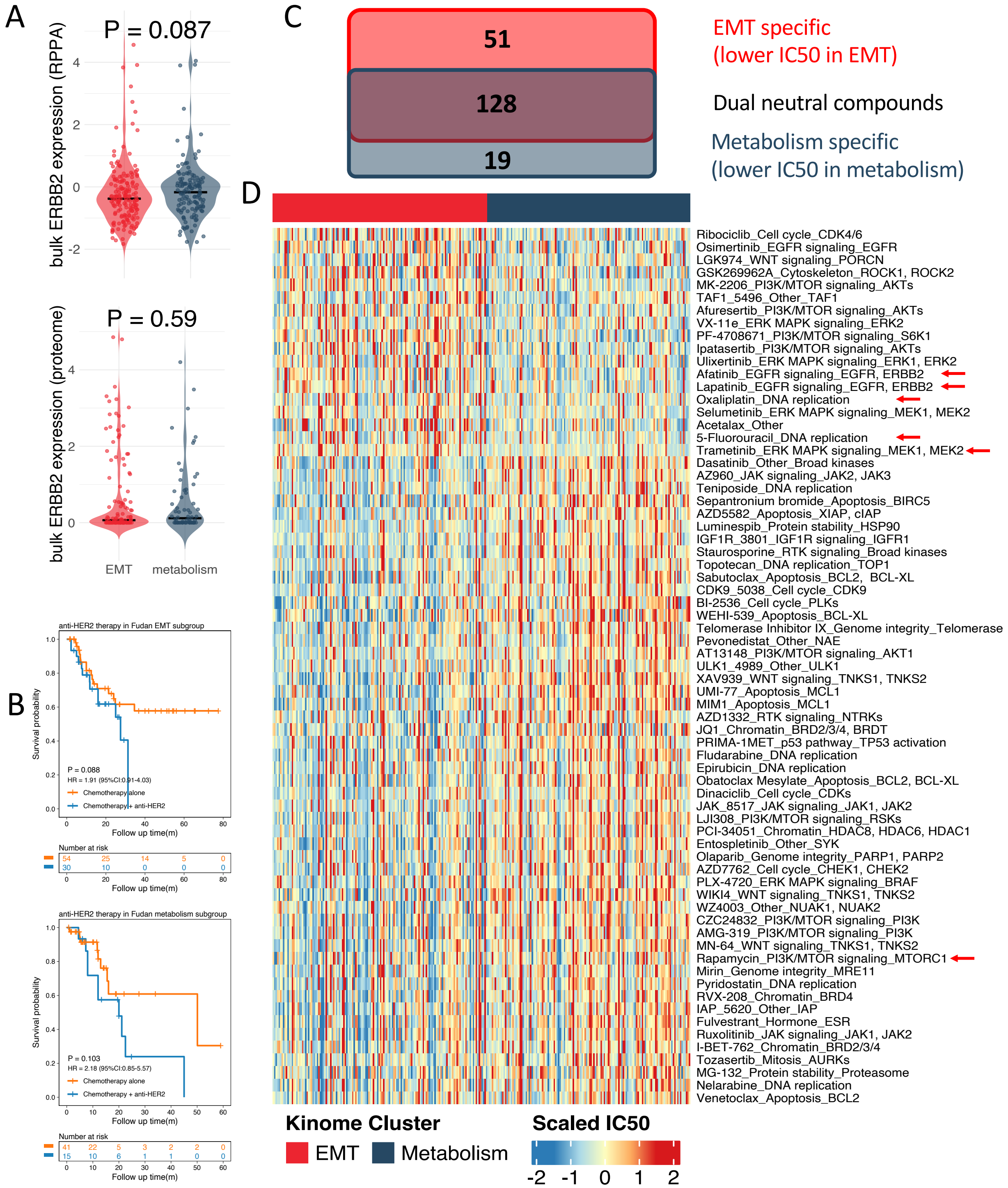

Figure S7 Predicted subgroup-specific drugs between EMT and metabolism subgroups
A Bulk ERBB2 protein expression across the kinomic subgroups in TCGA Reverse Phase Protein Array (RPPA) dataset (left) and Fudan proteomics dataset (right). **B** Stratification of the Fudan cohort using the 11-kinase signature. The survival difference, HRs and 95% CI between patients receiving neoadjuvant chemotherapy alone or in combination with anti-ERBB2 therapy were evaluated by univariate Cox proportional hazards regression analysis for the EMT (top) and metabolism (bottom) subgroups. **C** The numbers of predicted EMT-specific, metabolism-specific, and dual-neutral compounds were summarized as Venn diagram. **D** Subgroup-selective drugs were predicted by the oncoPredict algorithm using ACRG gene expression data. The heatmap indicates the normalized predicted IC50 for

each compound (The drug name, corresponding pathway and targets are indicated by row). Only compounds with a log2 fold change of IC50 > 0.1 and a P value < 0.05 between the kinomic subgroups are included. Red represents high IC50 whereas navy blue represents low IC50. Subclassification of the ACRG cohort is shown at the top. The red arrows highlight the genes mentioned in the main text.

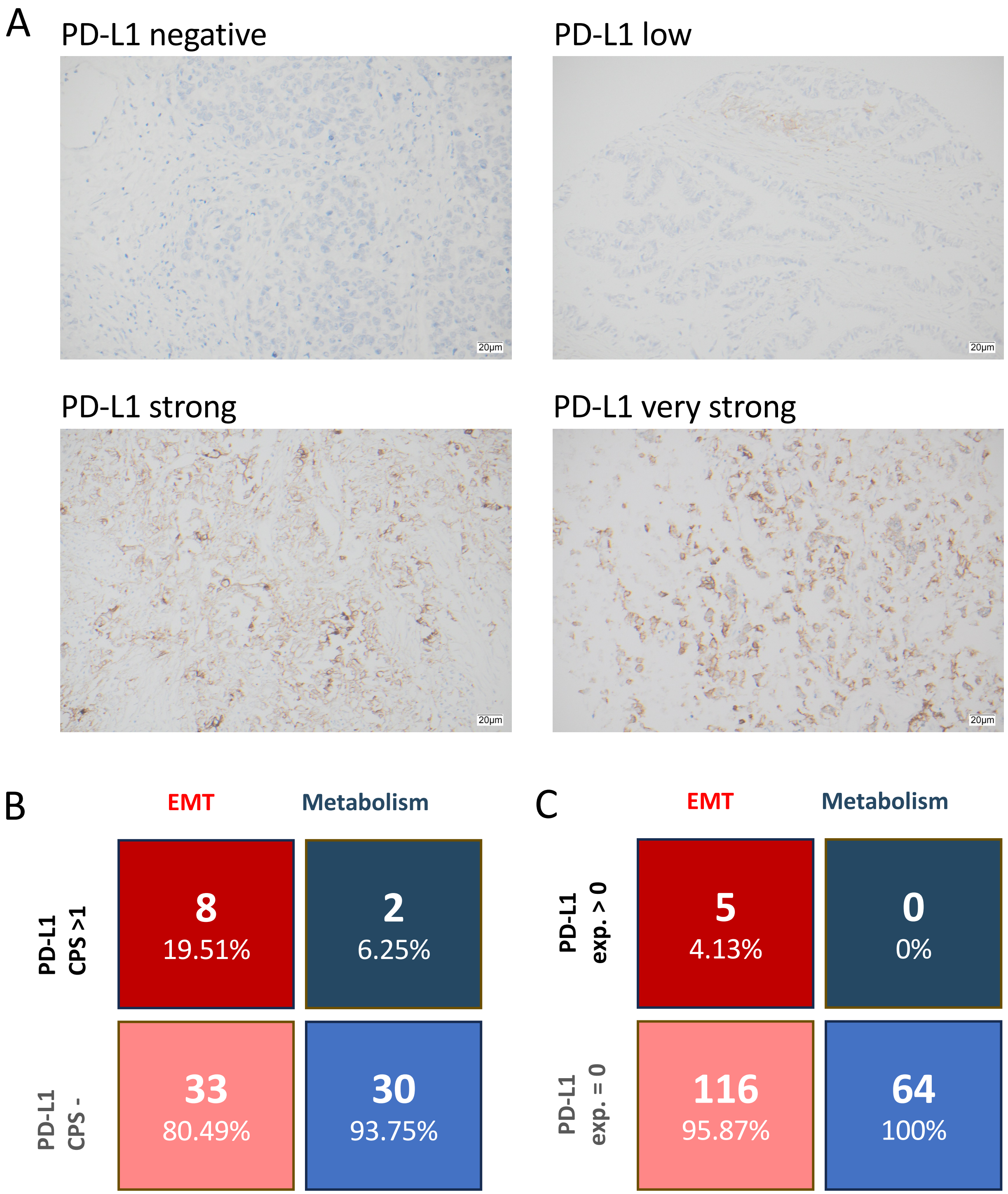

Figure S8 PD-L1 expression between EMT and metabolism subgroups

A Representative IHC staining of PD-L1 in gastric cancer from the Australian cohort, illustrating negative (CPS (Combined Positive Score) = 0), low (1 < CPS < 5), strong (5 < CPS < 10), and very strong (CPS > 10) PD-L1 expression levels. PD-L1 expression was primarily observed on tumour cells and/or tumour-infiltrating immune cells. **B** PD-L1 expression in the kinomic subgroup of the Australian cohort (Fisher's exact test). PD-L1 expression was assessed by IHC and scored by CPS. **C** PD-L1 expression in the kinomic subgroup of the Fudan cohort (Fisher's exact test). PD-L1 expression was assessed by Mass Spectrometry-based proteomics.

Table S2 and S6 were uploaded with separated Excel files.

Table S1. Baseline characteristics of patients from different cohorts

	TCGA	ACRG	KUGH	Australian	KUCM	PUCH	MDACC	Fudan
	RNA seq	Gene array	Gene array	Gene array	Gene array	Proteomics	Gene array	Proteomics
**Characteristic**	(N=375)	(N=300)	(N=93)	(N=68)	(N=109)	(N=82)	(N=40)	(N=206)
Age (year), median (IQR)	67 (15)	64 (15)	60 (19)	67 (18.25)	56 (15)	58.5 (16.75)	57.5 (21.5)	Not available
Sex								
Female (%)	134 (35.73%)	101 (33.67%)	20 (21.51%)	21 (30.88%)	40 (36.70%)	31 (37.80%)	13 (32.50%)	Not available
Male (%)	241 (64.27%)	199 (66.33%)	73 (78.49%)	47 (69.12%)	69 (63.30%)	51 (62.20%)	27 (67.50%)	Not available
TNM staging								Clinical TNM
I (%)	53 (14.13%)	30 (10.00%)	11 (11.83%)	12 (17.65%)	38 (34.86%)	5 (6.10%)	1 (2.50%)	0
II (%)	111 (29.60%)	97 (32.33%)	18 (19.35%)	15 (22.06%)	20 (18.35%)	20 (24.39%)	6 (15.00%)	32 (15.53%)
III (%)	150 (40.00%)	96 (32.00%)	27 (29.03%)	34 (50.00%)	36 (33.03%)	53 (64.63%)	12 (30.00%)	54 (26.21%)
IV (%)	38 (10.13%)	77 (25.67%)	36 (38.71%)	7 (10.29%)	15 (13.76%)	4 (4.88%)	21 (52.50%)	97 (47.09%)
Not available (%)	23 (6.13%)	0	1 (1.08%)	0	0	0	0	23 (11.17%)
Lauren classification								
Diffuse (%)	74 (19.73%)	135 (45.00%)	31 (33.33%)	29 (42.65%)	11 (10.09%)	82 (100%)	0	39 (18.93%)
Intestinal (%)	163 (43.47%)	146 (48.67%)	59 (63.44%)	27 (39.71%)	82 (75.22%)	0	0	95 (46.12%)
Mixed (%)	137 (36.53%)	19 (6.33%)	2 (2.15%)	11 (16.18%)	5 (4.59%)	0	0	54 (26.21%)
Not available (%)	1 (0.26%)	0	1 (1.08%)	1 (1.47%)	11 (10.09%)	0	40 (100.00%)	18 (8.74%)
Tumour location								
Cardia/Gastroesophageal junction (%)	90 (24.00%)	32 (10.67%)	7 (7.53%)	13 (19.12%)	0	20 (24.39%)	0	84 (40.78%)
Fundus/Body (%)	130 (34.67%)	115 (38.33%)	31 (33.33%)	42 (61.76%)	53 (48.62%)	33 (40.24%)	0	66 (32.04%)
Antrum (%)	139 (37.07%)	152 (50.67%)	55 (59.14%)	12 (17.65%)	56 (51.38%)	29 (35.37%)	0	54 (26.21%)
Not available (%)	16 (4.27%)	1 (0.33%)	0	1 (1.47%)	0	0	40 (100.00%)	2 (0.97%)
Chemotherapy								
Yes (%)	160 (42.67%)	144 (48.00%)	67 (72.04%)	35 (51.47%)	44 (40.37%)	65 (79.27%)	40 (100.00%)	188 (91.26%)
No (%)	215 (57.33%)	156 (52.00%)	26 (27.96%)	33 (48.53%)	65 (59.63%)	17 (20.73%)	0	18 (8.74%)
Radiotherapy								Anti-ERBB2
Yes (%)	65 (17.33%)	80 (26.67%)	0	27 (39.71%)	0	0	18 (45.00%)	70 (33.98%)
No (%)	310 (82.67%)	220 (73.33%)	93 (100.00%)	41 (60.29%)	109 (100.00%)	82 (100.00%)	22 (55.00%)	136 (66.02%)
Follow-up time (month), median (IQR)	14.63 (17.62)	57.85 (61.04)	33.80 (36.53)	29.18 (58.12)	47.00 (76.00)	22.77 (13.43)	16.40 (23.66)	12.00 (15.50)
Censored (%)	228 (60.80%)	148 (49.33%)	60 (64.52%)	16 (23.53%)	54 (49.54%)	60 (73.17%)	8 (20.00%)	145 (70.39%)
Events (%)	147 (39.20%)	152 (50.67%)	33 (35.48%)	52 (76.47%)	55 (50.46%)	22 (26.83%)	32 (80.00%)	61 (29.61%)

Table S3. A comparison between reassignment by 37-kinase signature and Lauren subclassification on TCGA cohort

Subtypes	EMT	Metabolism	P value
Diffuse (%)	42 (21.00%)	25 (16.13%)	0.18<br>Chi-square test
Intestinal (%)	79 (39.50%)	76 (49.03%)	
not otherwise specified (%)	79 (39.50%)	54 (34.84%)	

Table S4. Baseline patient characteristics and univariate analysis between meta subgroup of EMT and metabolism

Characteristic	Baseline comparison			Univariate analysis (Log-rank)		Univariate analysis (Cox proportional hazards model)					
	EMT	Metabolism	P value	EMT	Metabolism	EMT		Metabolism			
	(N=450)	(N=386)		P value	P value	HR (95%CI)	P value	HR (95%CI)	P value	RERI (95%CI)	P value
Age (year), median (IQR)	63 (17)	63 (17)	0.085	0.025	<0.0001	1.01 (1-1.02)	0.085	1.02 (1.01-1.04)	0.00037	-0.002 (-0.008-0.004)	0.73
Sex			0.33	0.64	0.89					-0.08 (-0.26-0.42)	0.32
Male (%)	300 (66.67%)	245 (63.47%)				Reference					
Female (%)	150 (33.33%)	141 (36.53%)				0.94 (0.71-1.23)	0.64	1.02 (0.76-1.38)	0.89		
TNM staging			0.81	<0.0001	<0.0001					-0.16 (-0.93-0.62)**	0.65
II (%)	145 (32.22%)	124 (32.12%)				Reference					
III (%)	204 (45.33%)	182 (47.15%)				1.79 (1.28-2.49)	0.00062	1.95 (1.33-2.87)	0.00069		
IV (%)	101 (22.44%)	80 (20.73%)				3.49 (2.44-5.00)	<0.0001	4.01 (2.66-6.06)	<0.0001		
Lauren classification			0.28	0.69	0.010					-0.07 (-0.41-0.27)***	0.66
Intestinal (%)	195 (43.33%)	165 (42.75%)				Reference					
Diffuse (%)	155 (34.44%)	147 (38.08%)				1.13 (0.84-1.5)	0.42	1.16 (0.84-1.62)	0.37		
Mixed (%)	77 (17.11%)	52 (13.47%)				1.12 (0.75-1.67)	0.58	1.97 (1.26-3.07)	0.0030		
Not available (%)	23 (5.11%)	22 (5.70%)				NA	NA	NA	NA		
Tumour location			0.83	0.62	0.33					-0.02 (-0.52-0.57)****	0.47
Cardia/Gastroesophageal junction (%)	71 (15.78%)	59 (15.28%)				Reference					
Fundus/Body (%)	166 (36.89%)	151 (39.12%)				0.87 (0.59-1.28)	0.48	0.83 (0.54-1.27)	0.40		
Antrum (%)	186 (41.33%)	155 (40.16%)				0.83 (0.56-1.21)	0.33	0.72 (0.47-1.12)	0.15		
Not available (%)	27 (6.00%)	21 (5.44%)				NA	NA	NA	NA		
Chemotherapy			0.77	0.00047	<0.0001					-0.25 (-0.58-0.09)	0.93
Yes (%)	231 (51.33%)	202 (52.33%)				0.63 (0.48-0.82)	0.00052	0.39 (0.29-0.53)	<0.0001		
No (%)	219 (48.67%)	184 (47.67%)				Reference					
Radiotherapy			0.82	0.080	<0.0001					-0.42 (-0.81-0.03)	0.98
Yes (%)	87 (19.33%)	77 (19.95%)				0.75 (0.54-1.04)	0.081	0.37 (0.24-0.56)	<0.0001		
No (%)	363 (80.67%)	309 (80.05%)				Reference					
Overall Survival (Month) median (95% CI*)	29.13 (25.97-39.00)	46.90 (34.37-70.20)	0.011								
Surgery Only OS (Month) median (95% CI*)	22.00 (16.83-27.97)	22.50 (17.43-33.27)	0.83								

* Kaplan–Meier estimator

** Estimated between stage IV and stage II-III

*** Estimated between intestinal and other types

**** Estimated between Cardia/Gatroesophageal junction and other locations

Table S5. A summary of reassignment on different cohorts by 11-kinase signature

Cohorts	Subtypes	EMT	Metabolism	P value
ACRG	MSI	43 (29.45%)	21 (15.22%)	<0.0001, Chi-Squared test
	MSS/EMT	33 (22.60%)	12 (8.70%)	
	MSS/TP53-	48 (32.88%)	56 (40.58%)	
	MSS/TP53+	22 (15.07%)	49 (35.51%)	
TCGA	CIN	72 (52.17%)	46 (49.46%)	0.75, Chi-Squared test
	EBV	11 (7.97%)	11 (11.83%)	
	GS	29 (21.01%)	17 (18.28%)	
	MSI	26 (18.84%)	19 (20.43%)	
Singapore	Invasive	34 (35.79%)	20 (21.74%)	0.0055, Chi-Squared test
	Metabolic	14 (14.74%)	31 (33.70%)	
	Proliferative	47 (49.47%)	41 (43.16%)	
KUGH	Mesenchymal	17 (36.96%)	4 (11.11%)	0.0078, Chi-Squared test
	Epithelial	29 (63.04%)	32 (88.89%)	

Table S7. A summary of tumour response rate to combination Anti-ERBB2 treatment for the EMT and metabolism subgroups

Subgroup	Anti-tumour treatment	Responder (CR+PR)	Non-responder (PD+SD)	P value
EMT	Anti-ERBB2 + Chemotherapy	18	10	0.036
	Chemotherapy	22	33	
Metabolism	Anti-ERBB2 + Chemotherapy	6	9	0.56
	Chemotherapy	21	22	

CR, complete response; PR, partial response; SD, stable disease; PD, progressive disease

References

1. Oh SC, Sohn BH, Cheong JH, Kim SB, Lee JE, Park KC, et al., Clinical and genomic landscape of gastric cancer with a mesenchymal phenotype. Nat Commun. 2018;9(1):1777.
2. Na D, Chae J, Cho SY, Kang W, Lee A, Min S, et al., Predictive biomarkers for 5-fluorouracil and oxaliplatin-based chemotherapy in gastric cancers via profiling of patient-derived xenografts. Nat Commun. 2021;12(1):4840.
