# Supplementary material for: Identification and validation of an 11-kinase signature that predicts chemo- and radiosensitivity in gastric cancer
Source: eBioMedicine. 2026 Feb 11;125:106154. doi: 10.1016/j.ebiom.2026.106154 (PMC12914827; doi:10.1016/j.ebiom.2026.106154)
Supplement: Reagent Validation File [file mmc4.docx]

Reagent Validation File

We are using the Dako 22c3 pham Dx IVD kit in accordance with the recommended manufacture protocol on the Dako omnis. The protocol is as follows:

Retrieval: Lo ph, citrate buffer

Primary time: 40 minutes

Blocking: 3 minutes

Mouse linker: 10 minutes

Polymer: HRP 20 minutes

Chromogen: 5 minutes

Counterstain: Haematoxylin 3 minutes

PD-L1 was initially validated using the Dako supplied and validated cell line control. Slides were run in accordance to manufacture recommended protocols. No parameters were changed. Slides were assessed by Prof. C. Mclean. We have subsequently validated in house control tissue using tonsil, known positive and negative staining tumour tissue types. The protocol is bi-annually assessed by external QAP programs.
